# Comparison of regional anesthetic techniques for postoperative analgesia after adult cardiac surgery: bayesian network meta-analysis

**DOI:** 10.3389/fcvm.2023.1078756

**Published:** 2023-05-22

**Authors:** Ke Zhou, Dongyu Li, Guang Song

**Affiliations:** ^1^ Department of Cardiac Surgery, Shengjing Hospital of China Medical University, Shenyang, China; ^2^ Department of Ultrasound, Shengjing Hospital of China Medical University, Shenyang, China

**Keywords:** cardiac surgery, postoperative pain, regional anesthesia, meta-analysis, network

## Abstract

**Background:**

Patients usually suffer acute pain after cardiac surgery. Numerous regional anesthetic techniques have been used for those patients under general anesthesia. The most effective regional anesthetic technique was still unclear.

**Methods:**

Five databases were searched, including PubMed, MEDLINE, Embase, ClinicalTrials.gov, and Cochrane Library. The efficiency outcomes were pain scores, cumulative morphine consumption, and the need for rescue analgesia in this Bayesian analysis. Postoperative nausea, vomiting and pruritus were safety outcomes. Functional outcomes included the time to tracheal extubation, ICU stay, hospital stay, and mortality.

**Results:**

This meta-analysis included 65 randomized controlled trials involving 5,013 patients. Eight regional anesthetic techniques were involved, including thoracic epidural analgesia (TEA), erector spinae plane block, and transversus thoracic muscle plane block. Compared to controls (who have not received regional anesthetic techniques), TEA reduced the pain scores at 6, 12, 24 and 48 h both at rest and cough, decreased the rate of need for rescue analgesia (OR = 0.10, 95% CI: 0.016–0.55), shortened the time to tracheal extubation (MD = −181.55, 95% CI: −243.05 to −121.33) and the duration of hospital stay (MD = −0.73, 95% CI: −1.22 to −0.24). Erector spinae plane block reduced the pain score 6 h at rest and the risk of pruritus, shortened the duration of ICU stay compared to controls. Transversus thoracic muscle plane block reduced the pain scores 6 and 12 h at rest compared to controls. The cumulative morphine consumption of each technique was similar at 24, 48 h. Other outcomes were also similar among these regional anesthetic techniques.

**Conclusions:**

TEA seems the most effective regional postoperative anesthesia for patients after cardiac surgery by reducing the pain scores and decreasing the rate of need for rescue analgesia.

**Systematic Review Registration:**

https://www.crd.york.ac.uk/prospero/, ID: CRD42021276645

## 1. Introduction

According to the latest cardiac surgery market report, approximately 900,000 cardiac surgeries are performed each year in the USA. Patients usually suffer acute pain after coronary artery bypass graft or valve surgery: 78% of patients experienced pain during coughing, while 49% of patients experienced pain at rest (1). It is imperative that we should take this pain seriously and treat promptly. Because inadequately controlled acute pain was associated with chronic pain and persistent postoperative pain (2). Meantime, increasing morbidity and mortality were associated with pain following cardiac surgery (3).

Although intravenous opioid is the first-line postoperative analgesic, opioid-related adverse effects, such as postoperative nausea and vomiting (PONV), pruritus, ventilator-associated pneumonia, can lead to other problems including prolonged intubation, and higher mortality (4). Numerous regional anesthetic techniques, such as thoracic epidural analgesia (TEA), paravertebral block (PVB), erector spinae plane block (ESPB), serratus anterior plane block (SAPB), pectoral nerve block (PECS), parasternal intercostal nerve block (PINB), transversus thoracic muscle plane block (TTMPB) and pecto-intercostal fascial block (PIFB) were introduced as a part of multimodal post-cardiac surgery analgesia, attempting to reduce the cumulative postoperative opiate consumption and pain scores. Few randomized controlled trials (RCTs) have compared these eight techniques. It remains uncertain which is the best technique for postoperative analgesia for cardiac surgery patients.

Network meta-analysis (NMA) pools evidence from a large number of comparisons and patients to compare several interventions, allowing for indirect comparisons and ranking. Therefore, we conducted this NMA with the aim to compare regional anesthetic techniques efficacy and safety for pain relief after cardiac surgery.

## 2. Materials and methods

The pre-registered protocol was implemented in the PROSPERO database (CRD42021276645). This paper was reported in accordance with PRISMA guideline.

### 2.1. Search strategy

On September 2, 2022, two investigators searched PubMed, MEDLINE, Embase, ClinicalTrials.gov, and Cochrane Library for relevant studies with the words “cardiac surgery/cardiac surgical procedures” and (“thoracic epidural analgesia”, or “paravertebral block”, or “erector spinae plane block”, or “serratus anterior plane block”, or “pectoral nerve block”, or “parasternal intercostal nerve block”, or “transversus thoracic muscle plane block”, or “pecto-intercostal fascial block”). Additionally, we read the references of articles in search for literature that met the criteria.

### 2.2. Study selection and data exclusion

Original studies were eligible if the following criteria were met: (i) RCT study in English; and (ii) assessed the efficacy and safety of regional postoperative anesthetic techniques after cardiac surgery under general anesthesia. Original studies were ineligible if the following criteria were met: (i) studies involving combination blocks (i.e., ESPB combined with PECS) (5); (ii) participants were children or animal.

The first author, year of publication, country, surgery type, anesthesia technique, groups and number of participants in each group, drug and dose for regional anesthetics, block timing, postoperative analgesia, and outcomes were extracted in the involved eligible studies. When the data could not be gathered from tables and full text, GetData Graph Digitizer (v 2.26) was used to obtain the numerical data from figures (6).

### 2.3. Outcomes

Efficiency outcomes were pain scores, the cumulative morphine consumption, and the need for rescue analgesia. Pain scores in the involved studies were converted to a standardized 0–100-point value (where 0 = no pain and 100 = worst pain imaginable) for statistics (6, 7). Five time points (2–4, 6, 12, 24, and 48 h after postoperative tracheal extubation) were selected. Any opiate medications other than intravenous morphine were converted to equivalents of morphine as our previous study (6). PONV and pruritus were safety outcomes. Functional outcomes included the time to tracheal extubation (min), intensive care uinit (ICU) stay (h), hospital stay (days), and mortality (in hospital) as the previous study (8).

### 2.4. Statistical analysis

Cochrane Collaboration's tool was used to evaluate the quality of involved studies. Mean difference (MD) and 95% confidence interval (CI) were used to report the cumulative morphine consumption, pain scores, the time to tracheal extubation, ICU stay, and hospital stay. Odds ratios (ORs) were used to report the risk of PONV, pruritus, mortality, and the need for rescue analgesia. In this Bayesian NMA, random-effects and consistency models were used to analyze data (four chains, 50,000 iterations, 20,000 per chain). Inconsistency was assessed by the node-splitting method with Bayesian *P*-value. The surface under the cumulative ranking curve (SUCRA) was calculated and ranked. Begg's and Egger's tests were performed to evaluate publication bias. All analyses were conducted using the “gemtc” package of R version 4.0.2 (R Foundation, Vienna, Austria).

## 3. Results

### 3.1. Baseline characteristics of included studies

**Figure 1:**
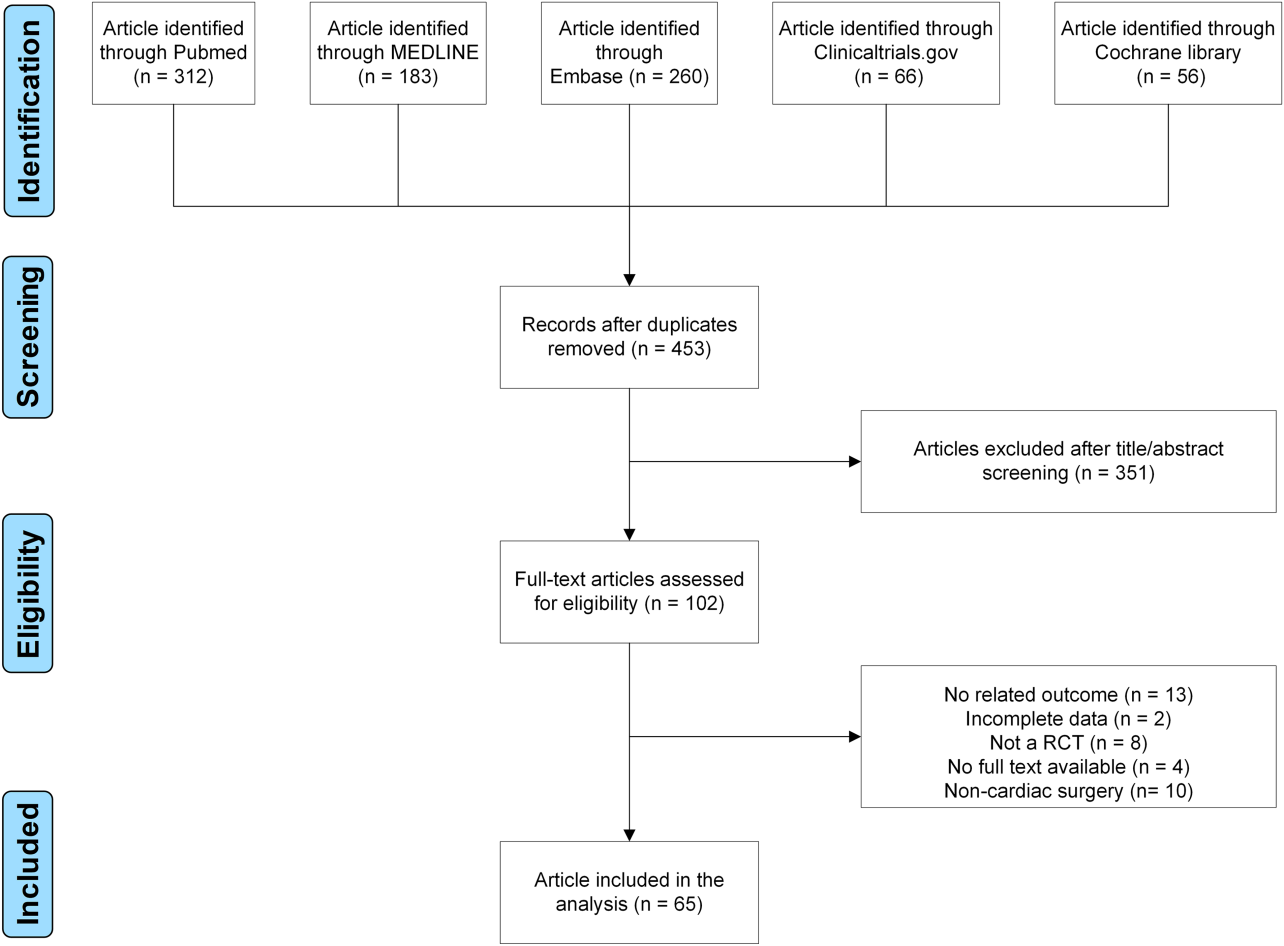
Flow-chart of study selection.

Finally, 65 RCTs were included using our search strategy (Supplementary Table S1 and Figure 1) (9–73). Assessment of bias risk is demonstrated (Supplementary Figures S1,S2). In the period from 1987 to 2021, 65 RCTs were carried out, involving 5,013 participants (Supplementary Table S2). Eight techniques were evaluated, including ESPB, PECS, PIFB, PINB, PVB, SAPB, TEA, and TTMPB (Supplementary Figure S3). 72.3% (47/65) studies involved coronary artery bypass graft; 23.1% (15/65) involved mixed surgery; 4.6% (3/65) involved valve surgery. In total, sixty-four studies were two-arm, and only one study was three-arm and published in 2020. Drugs, dose, block timing, postoperative analgesia, and outcomes are also shown in Supplementary Table S2.

### 3.2. Efficiency outcomes

**Figure 2:**
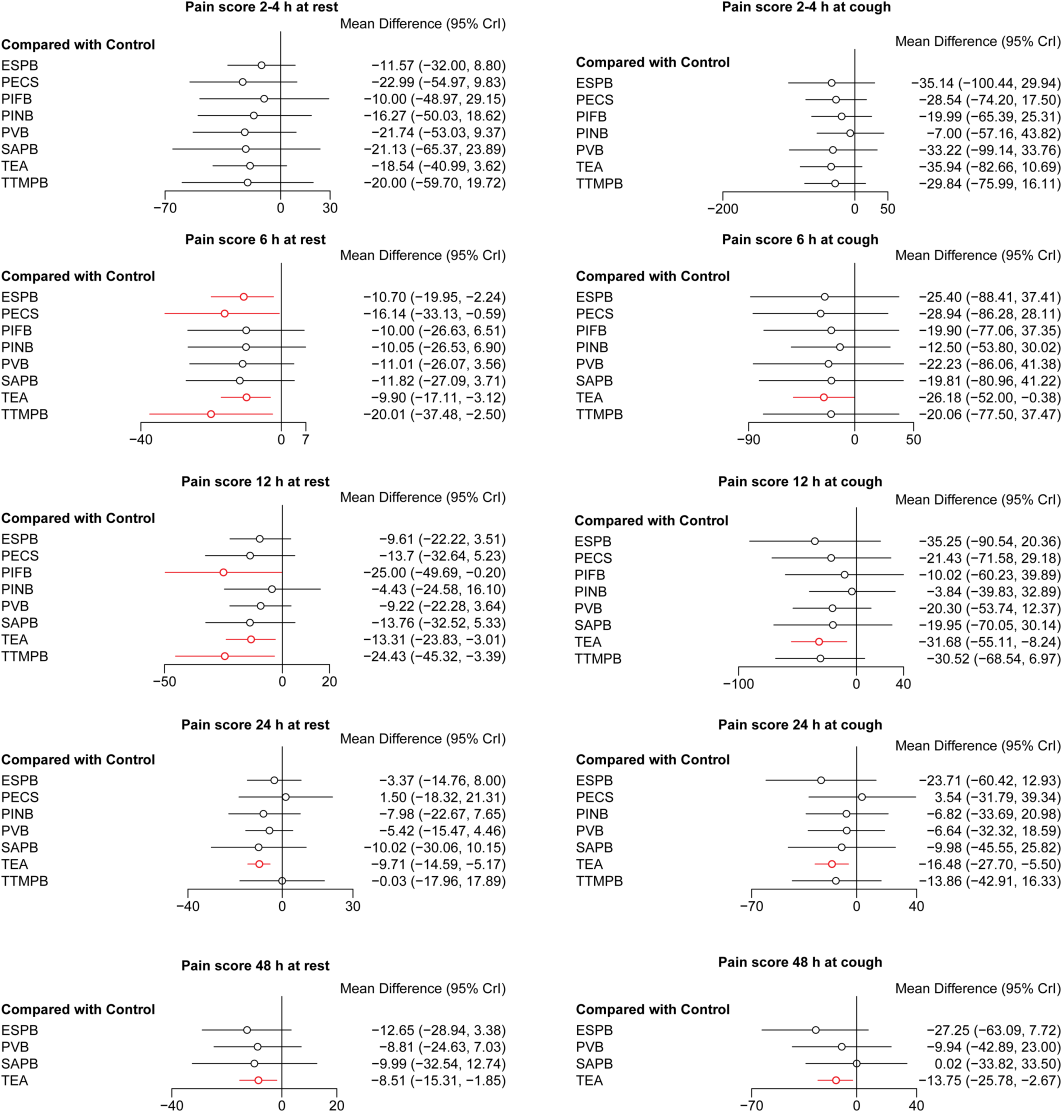
Forest plots of pain scores. The results with a *p*-value <0.05 are marked in red. ESPB, erector spinae plane block; PECS, pectoral nerve block; PIFB, pecto-intercostal fascial block; PINB, parasternal intercostal nerve block; PVB, paravertebral block; SAPB, serratus anterior plane block; TEA, thoracic epidural analgesia; TTMPB, transversus thoracic muscle plane block.

There was no difference in pain scores at 2–4 h both at rest and cough among these regional anesthetic techniques. Pain scores at 6, 12, 24, 48 h both at rest and cough were lower for TEA than for controls (Figure 2). Pain scores at 6, 12 h at rest were lower for TTMPB than for controls. Pain scores at 6 h at rest were lower for ESPB and PECS than for controls. Pain score at 12 h at rest was lower for PIFB than for controls. Pain scores were similar among PINB, PVB, SAPB, and controls. Pairwise comparisons are shown in Supplementary Tables S3–S12.

**Figure 3:**
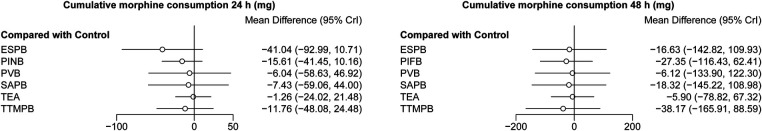
Forest plots of cumulative morphine consumption at 24 and 48 h. The results with a *p*-value <0.05 are marked in red. ESPB, erector spinae plane block; PIFB, pecto-intercostal fascial block; PINB, parasternal intercostal nerve block; PVB, paravertebral block; SAPB, serratus anterior plane block; TEA, thoracic epidural analgesia; TTMPB, transversus thoracic muscle plane block.

**Figure 4:**
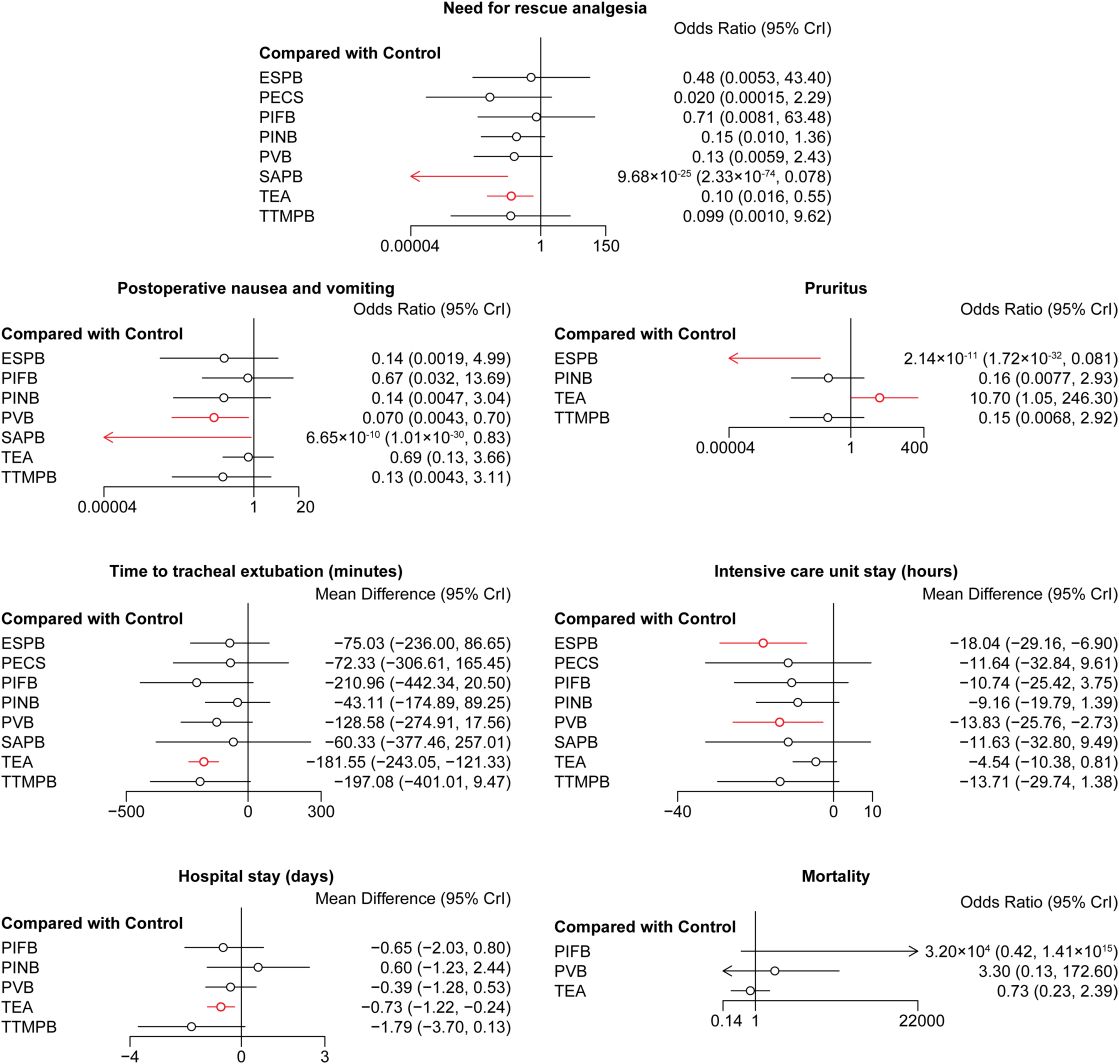
Forest plots of other outcomes. The results with a *p*-value <0.05 are marked in red. ESPB, erector spinae plane block; PECS, pectoral nerve block; PIFB, pecto-intercostal fascial block; PINB, parasternal intercostal nerve block; PVB, paravertebral block; SAPB, serratus anterior plane block; TEA, thoracic epidural analgesia; TTMPB, transversus thoracic muscle plane block.

There was no difference in the cumulative morphine consumption at 24, 48 h among these regional anesthetic techniques (Figure 3). SAPB and TEA reduced the rate of need for rescue analgesia compared with controls (OR = 9.68 × 10^−25^, 95% CI: 2.33 × 10^−74^–0.078; OR = 0.10, 95% CI: 0.016–0.55, respectively, Figure 4). Pairwise comparisons are shown in Supplementary Tables S13–S15.

### 3.3. Safety outcomes

SAPB and PVB reduced the risk of PONV compared with the control group (OR = 6.65 × 10^−10^, 95% CI: 1.01 × 10^−30^–0.83; OR = 0.070, 95% CI: 0.0043–0.70, respectively, Figure 4). ESPB reduced the risk of pruritus compared with controls (OR = 2.14 × 10^−11^, 95% CI: 1.72 × 10^−32^–0.081, Figure 4). TEA increased the risk of pruritus compared with the control group (OR = 10.70, 95% CI: 1.05–246.30, Figure 4). Pairwise comparisons are shown in Supplementary Tables S16,S17.

### 3.4. Functional outcomes

TEA shortened the time to tracheal extubation compared with controls (MD = −181.55, 95% CI: −243.05 to −121.33, Figure 4). ESPB and PVB shortened the ICU stay compared with controls (MD = −18.04, 95% CI: −29.16 to −6.90; MD = −13.83, 95% CI: −25.76 to −2.73, respectively, Figure 4). TEA shortened the hospital stay compared with controls (MD = −0.73, 95% CI: −1.22 to −0.24, Figure 4). There was no difference in the mortality among these regional anesthetic techniques. Pairwise comparisons are shown in Supplementary Tables S18–S21.

### 3.5. Inconsistency, ranking, certainty of evidence, and publication bias

**Figure 5:**
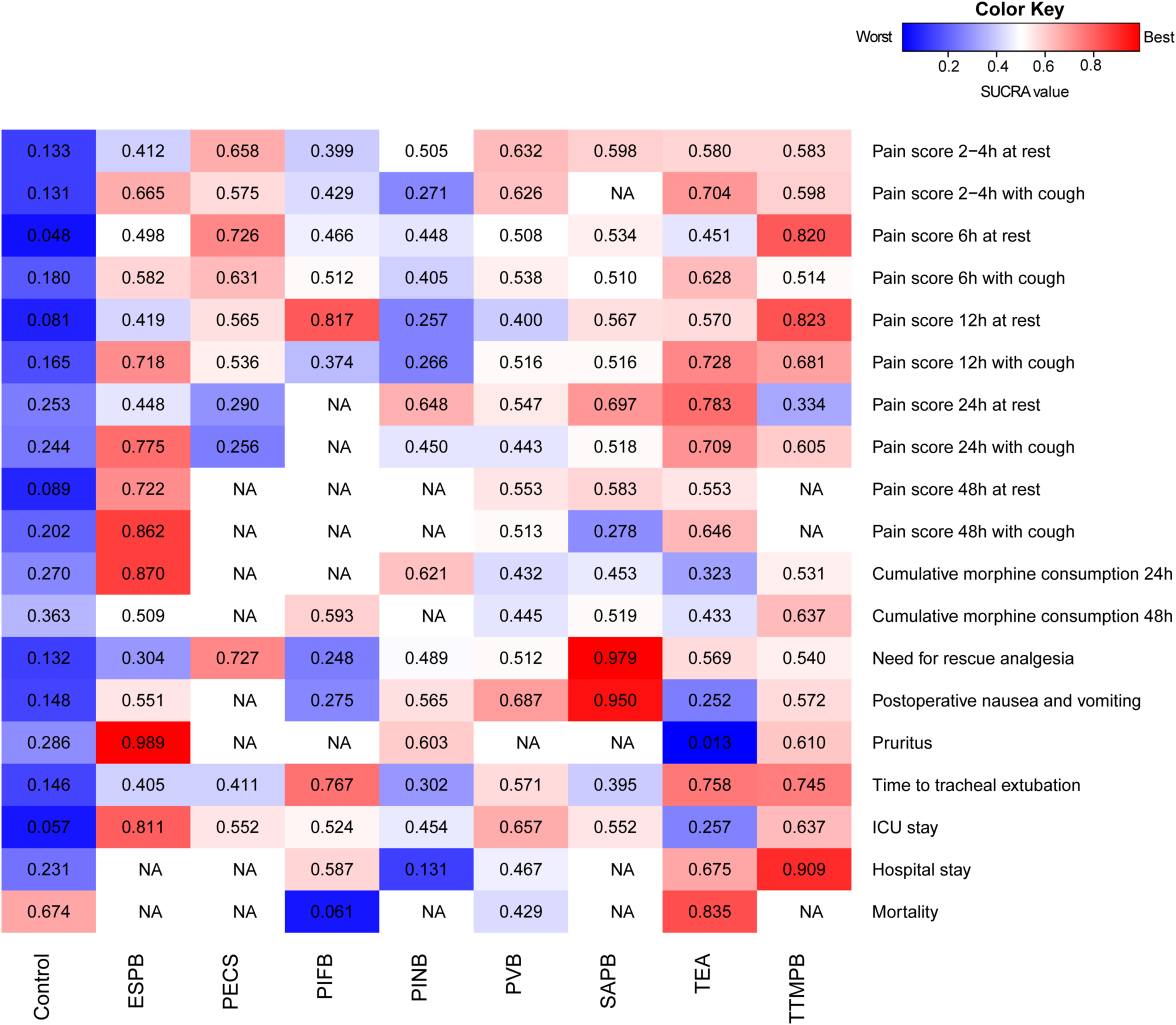
Heat maps of regional anesthetic techniques studied in adult patient with cardiac surgery under general anesthesia for 19 outcomes. Each box is colored according to the SUCRA value of the corresponding anesthetic technique and outcome. Uncolored boxes labeled NA mean that outcome was not involved in the underlying treatment. ESPB, erector spinae plane block; ICU, intensive care unit; NA, not applicable; PECS, pectoral nerve block; PIFB, pecto-intercostal fascial block; PINB, parasternal intercostal nerve block; PVB, paravertebral block; SAPB, serratus anterior plane block; SUCRA, surface under the cumulative ranking curve; TEA, thoracic epidural analgesia; TTMPB, transversus thoracic muscle plane block.

There was a significance level of *P* > 0.05 for all cases, indicating inconsistencies were not sufficient to influence the conclusions of our NMA (Supplementary Figures S4,S5). The ranks of each outcome are shown in Figure 5. The certainty of evidence is shown in Table 1. The assessment of publication bias is revealed in Supplementary Table S22.

**Table 1:** Summary of the results of NMA and GRADE quality score assessment for the outcomes.

	Study number	Participants number	Conclusion	GRADE quality score
**Efficiency outcomes**				
Pain score 2–4 h at rest	13	626	No regional anesthetic technique superior to the controls	Low^ab^
Pain score 2–4 h at cough	7	285	No regional anesthetic technique superior to the controls	Moderate^a^
Pain score 6 h at rest	17	859	TEA, ESPB, PECS, and TTMPB superior to the controls	Moderate^a^
Pain score 6 h at cough	13	695	TEA superior to the controls	Moderate^a^
Pain score 12 h at rest	20	1,080	TEA, PIFB, and TTMPB superior to the controls	Moderate^a^
Pain score 12 h at cough	15	771	TEA superior to the controls	Moderate^a^
Pain score 24 h at rest	29	2,164	TEA superior to the controls	Moderate^a^
Pain score 24 h at cough	19	1,139	TEA superior to the controls	Moderate^a^
Pain score 48 h at rest	15	1,579	TEA superior to the controls	Moderate^a^
Pain score 48 h at cough	12	810	TEA superior to the controls	Low^ab^
Cumulative morphine consumption 24 h (mg)	14	705	No regional anesthetic technique superior to the controls	Low^ab^
Cumulative morphine consumption 48 h (mg)	9	536	No regional anesthetic technique superior to the controls	Low^ab^
Need for rescue analgesia	19	1,297	SAPB and TEA superior to control; SAPB superior to TEA	Moderate^a^
**Safety outcomes**				
PONV	11	752	SAPB and PVB superior to control	Moderate^a^
Pruritus	5	280	ESPB superior to control, PINB and TTMPB; PINB, TTMPB, and control superior to TEA	Moderate^a^
**Functional outcomes**				
Time to tracheal extubation (minutes)	53	4,080	TEA superior to control	Low^ab^
ICU stay (hours)	26	2,486	ESPB and PVB superior to control	Low^ab^
Hospital stay (days)	27	2,640	TEA superior to control	Low^ab^
Mortality	13	1,960	No regional anesthetic technique superior to the controls	Moderate^a^

ESPB, erector spinae plane block; ICU, intensive care unit; NA, not applicable; PECS, pectoral nerve block; PIFB, pecto-intercostal fascial block; PINB, parasternal intercostal nerve block; PONV, postoperative nausea and vomiting; PVB, paravertebral block; SAPB, serratus anterior plane block; TEA, thoracic epidural analgesia; TTMPB, transversus thoracic muscle plane block.

^a^ Rated down for concerns related to imprecision.

^b^ Rated down for concerns related to publication bias.

## 4. Discussion

This paper is the first comprehensive NMA assessing the efficacy and safety of regional anesthetic techniques after cardiac surgery. Though none of the regional anesthetic techniques could reduce the cumulative morphine consumption compared with controls, our results revealed that TEA, ESPB, and TTMPB reduced pain scores at different time points. TEA also reduced the rate of need for rescue analgesia, the time to tracheal extubation, and duration of hospital stay. ESPB could reduce the risk of pruritus and shorten the duration of ICU stay.

Pain is the most severe during the first 24 h following cardiac surgery, and it gradually decreases (74). Ineffective postoperative pain management may cause immunosuppression, infections, and less effective wound healing (75). For most people, their first exposure to opioids was during surgeries (76). Regional anesthetic techniques may play a role in decreasing or sparing opioid exposure when opioid dependence, opioid overdose, and community overuse and misuse are becoming incisive social issues. However, currently there is no consensus on the best regional anesthetic technique. Our results suggested TEA may be the most effective regional anesthetic technique for cardiac surgery patients under general anesthesia.

In 1954, TEA was first introduced in cardiac surgery (77). This regional anesthetic technique blocks thoracic spinal cord (T1–T5). Our NMA has once again confirmed that TEA provided superior analgesia compared with controls by reducing the pain score and the rate of need for rescue analgesia (78, 79). TEA could shorten the time to tracheal extubation, which made a step further from the goal of early mobilization. Previous study revealed mobilization reduced the risk of postoperative pulmonary thromboembolism and other complications (80). TEA was recommended as Class IIb treatment according to the guideline in 2017 (81). Meantime, a previous meta-analysis revealed TEA reduced the risk of perioperative myocardial infarction, respiratory depression, and atrial fibrillation/flutter for cardiac surgery patients (78). Our results showed a similar mortality between TEA and controls, which was consistent with the previous study (78). Whereas, a study with transapical transcatheter aortic valve implantation revealed TEA provided superior analgesia following and decreased one-year mortality (82). The changes in long-term mortality need to be further studied. As the pain was relieved, some adverse effects began to appear. The most frequently discussed complication was epidural hematoma (83). Because of the low incidence rate (incidence of 1:3,552–12,000), none of our involved study reported this complication when performing TEA (83, 84). In our NMA, we found TEA increased the risk of pruritus which is an opioid-related side effect. The cardiac anesthesiologists should pay attention to these two adverse effects when performed TEA.

It is likely that PVB is the most commonly administered paraxial nerve block (85). PONV in cardiac surgery affects 20%–67% of all patients, increases adrenergic stimulation, limits mobility and oral intake, and is distressing to the patient (86). 18.4% of patients who used opioids under cardiac surgery suffered pruritus (87). In our NMA, we found PVB reduced the risk of PONV. Similar results were reported by two meta-analyses, suggesting PVB had reduced the incidence of PONV as postcardiothoracic surgery analgesia when compared with TEA (88, 89). ESPB, as an ultrasound-guided PVB variant, was first described in 2016 (90). ESPB blocked dorsal and ventral rami of spinal nerve roots as same as PVB (85, 91). ESPB not only had a greater analgesic benefit in the first six hours after tracheal extubation, but also reduced the risk of pruritus in our analysis. However, due to limited numbers of trials, the efficacy and safety profile of ESPB require further investigation.

PINB, TTMPB, and PIFB complement the anteromedial chest wall by providing anesthesia confined to the parasternal region. According to the nuance of the injection position, they were divided into two categories: regional anesthetics was injected between the internal intercostal and pectoralis major muscles in PIFB and PINB; regional anesthetics was deeply injected between the internal intercostal and transverse thoracic muscles in TTMPB (3, 91, 92). All these three techniques anesthetized the anterior branches of the intercostal nerves. PINB, also known as parasternal block, was first described for cardiac surgery in 2005 (30). When PINB was performed, regional anesthetics was usually given for five interspaces bilaterally, over the periosteum, and/or around the mediastinal tubes. Under ultrasound guidance, the patients with PIFB received regional anesthetics bilaterally at the target sites for breast surgery since 2014 (93). TTMPB became an analgesic block for cardiac surgery soon afterward it was described as an adjunct of PECS during breast surgery in 2015 (94). Because of the similarities of PINB, TTMPB, and PIFB mentioned above, their anesthetic efficiency and safety were also similar in our analysis. Some researchers believed that PINB and PIFB were the same blocks (95). Some authors promoted PIFB because of easily identify with ultrasound (91). Although pain scores at 6, 12 h at rest were lower for TTMPB, evidence for cardiac surgery patients is extremely limited.

On the anterolateral chest wall, a new method named “PECS” was reported to anesthetize the medial-lateral pectoral nerves, long thoracic nerve, and thoracodorsal nerve since 2011 (96). In the similar region, SAPB was established which could block the anterior and lateral cutaneous branches of the intercostal nerves. Our results revealed SAPB reduced the rate of need for rescue analgesia compared with TEA, and PECS had lower pain score at 6 h at rest compared with controls. As PECS and SAPB don't cause sympathectomy and can be performed in patients on anticoagulants, they are the options for patients with contraindications for PVB or TEA (97).

### 4.1. Limitation

First, the results regarding to the emerging techniques, including TTMPB, PIFB, ESPB, PECS, and SAPB, need to be confirmed in more RCTs. Several ongoing trials with regional anesthetic techniques for cardiac surgery were shown in Supplementary Table S23. The results of mentioned techniques may be affected by publication bias, suggesting that future meta-analyses may draw different conclusions on some outcomes we analyzed. Second, surgical techniques, perioperative care protocols, local anesthetics, and postoperative adjunctive analgesia may be underlying confounders that were not adjustable. Meanwhile, not all of the RCTs clearly stated that multimodal postoperative analgesia was utilized. Regional anesthesia modalities should be regarded as complementary rather than an alternative to a multimodal analgesic strategy (8, 98). Third, not all time points of pain score or cumulative morphine consumption were assessed in each involved study. Fourth, wound infusion was not involved in our analysis because wound infusion was not recommended in cardiac surgery in the guideline of European Association of Cardio-Thoracic Surgery (81). Fifth, some safety outcomes (hematoma, wound infection, sedation, and urinary retention) and cardiac functional outcomes (myocardial infarction, arrhythmia, and supraventricular tachycardia) were limited and excluded in this NMA. Fourth, all blocks in the involved studies were performed before operation or after operation. None of previous studies reported there was potential differences between these groups.

## 5. Conclusions

TEA seems the most effective regional postoperative anesthesia for patients after cardiac surgery by reducing pain scores and decreasing the rate of need for rescue analgesia.

## Data Availability

The original contributions presented in the study are included in the article/**Supplementary Material**, further inquiries can be directed to the corresponding author.

## References

[1] Lahtinen P Kokki H Hynynen M. Pain after cardiac surgery: a prospective cohort study of 1-year incidence and intensity. Anesthesiology. (2006) 105:794–800. 10.1097/00000542-200610000-00026 17006079

[2] Echeverria-Villalobos M Stoicea N Todeschini AB Fiorda-Diaz J Uribe AA Weaver T Enhanced recovery after surgery (ERAS): a perspective review of postoperative pain management under ERAS pathways and its role on opioid crisis in the United States. Clin J Pain. (2020) 36:219–26. 10.1097/AJP.0000000000000792 31868759

[3] Caruso TJ Lawrence K Tsui BCH. Regional anesthesia for cardiac surgery. Curr Opin Anaesthesiol. (2019) 32:674–82. 10.1097/ACO.0000000000000769 31356362

[4] Liu J Zhang S Chen J Mao Y Shao X Li Y Risk factors for ventilator-associated events: a prospective cohort study. Am J Infect Control. (2019) 47:744–9. 10.1016/j.ajic.2018.09.032 30584021

[5] Gaweda B Borys M Belina B Bak J Czuczwar M Woloszczuk-Gebicka B Postoperative pain treatment with erector spinae plane block and pectoralis nerve blocks in patients undergoing mitral/tricuspid valve repair - a randomized controlled trial. BMC Anesthesiol. (2020) 20:51. 10.1186/s12871-020-00961-8 32106812 PMC7047405

[6] Wang J Zhao G Song G Liu J. The efficacy and safety of local anesthetic techniques for postoperative analgesia after cesarean section: a Bayesian network meta-analysis of randomized controlled trials. J Pain Res. (2021) 14:1559–72. 10.2147/JPR.S313972 34103981 PMC8180269

[7] Mercier F Claret L Prins K Bruno R. A model-based meta-analysis to compare efficacy and tolerability of tramadol and tapentadol for the treatment of chronic non-malignant pain. Pain Ther. (2014) 3:31–44. 10.1007/s40122-014-0023-5 25135386 PMC4108025

[8] Wong HY Pilling R Young BWM Owolabi AA Onwochei DN Desai N. Comparison of local and regional anesthesia modalities in breast surgery: a systematic review and network meta-analysis. J Clin Anesth. (2021) 72:110274. 10.1016/j.jclinane.2021.110274 33873002

[9] El-Baz N Goldin M. Continuous epidural infusion of morphine for pain relief after cardiac operations. J Thorac Cardiovasc Surg. (1987) 93:878–83. 10.1016/S0022-5223(19)37048-5 2952842

[10] Rein KA Stenseth R Myhre HO Levang OW Krogstad A. The influence of thoracic epidural analgesia on transcapillary fluid balance in subcutaneous tissue. A study in patients undergoing aortocoronary bypass surgery. Acta Anaesthesiol Scand. (1989) 33:79–83. 10.1111/j.1399-6576.1989.tb02865.x 2644753

[11] Liem TH Hasenbos MA Booij LH Gielen MJ. Coronary artery bypass grafting using two different anesthetic techniques: part 2: postoperative outcome. J Cardiothorac Vasc Anesth. (1992) 6:156–61. 10.1016/1053-0770(92)90190-I 1533166

[12] Stenseth R Bjella L Berg EM Christensen O Levang OW Gisvold SE. Effects of thoracic epidural analgesia on pulmonary function after coronary artery bypass surgery. Eur J Cardiothorac Surg. (1996) 10:859–65; discussion 866. 10.1016/S1010-7940(96)80311-3 8911839

[13] Fawcett WJ Edwards RE Quinn AC MacDonald IA Hall GM. Thoracic epidural analgesia started after cardiopulmonary bypass. Adrenergic, cardiovascular and respiratory sequelae. Anaesthesia. (1997) 52:294–9. 10.1111/j.1365-2044.1997.80-az0088.x 9135178

[14] Brix-Christensen V Tonnesen E Sorensen IJ Bilfinger TV Sanchez RG Stefano GB. Effects of anaesthesia based on high versus low doses of opioids on the cytokine and acute-phase protein responses in patients undergoing cardiac surgery. Acta Anaesthesiol Scand. (1998) 42:63–70. 10.1111/j.1399-6576.1998.tb05082.x 9527747

[15] Loick HM Schmidt C Van Aken H Junker R Erren M Berendes E High thoracic epidural anesthesia, but not clonidine, attenuates the perioperative stress response via sympatholysis and reduces the release of troponin T in patients undergoing coronary artery bypass grafting. Anesth Analg. (1999) 88:701–9. 10.1097/00000539-199904000-00001 10195508

[16] Tenling A Joachimsson PO Tyden H Wegenius G Hedenstierna G. Thoracic epidural anesthesia as an adjunct to general anesthesia for cardiac surgery: effects on ventilation-perfusion relationships. J Cardiothorac Vasc Anesth. (1999) 13:258–64. 10.1016/S1053-0770(99)90260-4 10392674

[17] Dhole S Mehta Y Saxena H Juneja R Trehan N. Comparison of continuous thoracic epidural and paravertebral blocks for postoperative analgesia after minimally invasive direct coronary artery bypass surgery. J Cardiothorac Vasc Anesth. (2001) 15:288–92. 10.1053/jcan.2001.23271 11426357

[18] Jidéus L Joachimsson PO Stridsberg M Ericson M Tyden H Nilsson L Thoracic epidural anesthesia does not influence the occurrence of postoperative sustained atrial fibrillation. Ann Thorac Surg. (2001) 72:65–71. 10.1016/S0003-4975(01)02631-5 11465233

[19] Scott NB Turfrey DJ Ray DA Nzewi O Sutcliffe NP Lal AB A prospective randomized study of the potential benefits of thoracic epidural anesthesia and analgesia in patients undergoing coronary artery bypass grafting. Anesth Analg. (2001) 93:528–35. 10.1097/00000539-200109000-00003 11524314

[20] Bach F Grundmann U Bauer M Buchinger H Soltesz S Graeter T Modulation of the inflammatory response to cardiopulmonary bypass by dopexamine and epidural anesthesia. Acta Anaesthesiol Scand. (2002) 46:1227–35. 10.1034/j.1399-6576.2002.461010.x 12421195

[21] de Vries AJ Mariani MA van der Maaten JM Loef BG Lip H. To ventilate or not after minimally invasive direct coronary artery bypass surgery: the role of epidural anesthesia. J Cardiothorac Vasc Anesth. (2002) 16:21–6. 10.1053/jcan.2002.29645 11854873

[22] Fillinger MP Yeager MP Dodds TM Fillinger MF Whalen PK Glass DD. Epidural anesthesia and analgesia: effects on recovery from cardiac surgery. J Cardiothorac Vasc Anesth. (2002) 16:15–20. 10.1053/jcan.2002.29639 11854872

[23] Priestley MC Cope L Halliwell R Gibson P Chard RB Skinner M Thoracic epidural anesthesia for cardiac surgery: the effects on tracheal intubation time and length of hospital stay. Anesth Analg. (2002) 94:275–82. 10.1213/00000539-200202000-00009 11812684

[24] Berendes E Schmidt C Van Aken H Hartlage MG Wirtz S Reinecke H Reversible cardiac sympathectomy by high thoracic epidural anesthesia improves regional left ventricular function in patients undergoing coronary artery bypass grafting: a randomized trial. Arch Surg. (2003) 138:1283–90; discussion 1291. 10.1001/archsurg.138.12.1283 14662525

[25] Royse C Royse A Soeding P Blake D Pang J. Prospective randomized trial of high thoracic epidural analgesia for coronary artery bypass surgery. Ann Thorac Surg. (2003) 75:93–100. 10.1016/S0003-4975(02)04074-2 12537199

[26] Volk T Dopfmer UR Schmutzler M Rimpau S Schnitzler H Konertz W Stress induced IL-10 does not seem to be essential for early monocyte deactivation following cardiac surgery. Cytokine. (2003) 24:237–43. 10.1016/S1043-4666(03)00090-5 14609565

[27] Kendall JB Russell GN Scawn ND Akrofi M Cowan CM Fox MA. A prospective, randomised, single-blind pilot study to determine the effect of anaesthetic technique on troponin T release after off-pump coronary artery surgery. Anaesthesia. (2004) 59:545–9. 10.1111/j.1365-2044.2004.03713.x 15144293

[28] Nygård E Sorensen LH Hviid LB Pedersen FM Ravn J Thomassen L Effects of amiodarone and thoracic epidural analgesia on atrial fibrillation after coronary artery bypass grafting. J Cardiothorac Vasc Anesth. (2004) 18:709–14. 10.1053/j.jvca.2004.08.006 15650978

[29] Barrington MJ Kluger R Watson R Scott DA Harris KJ. Epidural anesthesia for coronary artery bypass surgery compared with general anesthesia alone does not reduce biochemical markers of myocardial damage. Anesth Analg. (2005) 100:921–8. 10.1213/01.ANE.0000146437.88485.47 15781499

[30] McDonald SB Jacobsohn E Kopacz DJ Desphande S Helman JD Salinas F Parasternal block and local anesthetic infiltration with levobupivacaine after cardiac surgery with desflurane: the effect on postoperative pain, pulmonary function, and tracheal extubation times. Anesth Analg. (2005) 100:25–32. 10.1213/01.ANE.0000139652.84897.BD 15616047

[31] Hansdottir V Philip J Olsen MF Eduard C Houltz E Ricksten SE. Thoracic epidural versus intravenous patient-controlled analgesia after cardiac surgery: a randomized controlled trial on length of hospital stay and patient-perceived quality of recovery. Anesthesiology. (2006) 104:142–51. 10.1097/00000542-200601000-00020 16394700

[32] Bakhtiary F Therapidis P Dzemali O Ak K Ackermann H Meininger D Impact of high thoracic epidural anesthesia on incidence of perioperative atrial fibrillation in off-pump coronary bypass grafting: a prospective randomized study. J Thorac Cardiovasc Surg. (2007) 134:460–4. 10.1016/j.jtcvs.2007.03.043 17662790

[33] Barr AM Tutungi E Almeida AA. Parasternal intercostal block with ropivacaine for pain management after cardiac surgery: a double-blind, randomized, controlled trial. J Cardiothorac Vasc Anesth. (2007) 21:547–53. 10.1053/j.jvca.2006.09.003 17678782

[34] Kiliçkan L Yumuk Z Bayindir O. The effect of combined preinduction thoracic epidural anaesthesia and glucocorticoid administration on perioperative interleukin-10 levels and hyperglycemia. A randomized controlled trial. J Cardiovasc Surg. (2008) 49:87–93. 18212693

[35] Mehta Y Arora D Sharma KK Mishra Y Wasir H Trehan N. Comparison of continuous thoracic epidural and paravertebral block for postoperative analgesia after robotic-assisted coronary artery bypass surgery. Ann Card Anaesth. (2008) 11:91–6. 10.4103/0971-9784.41576 18603748

[36] Palomero Rodríguez MA Suarez Gonzalo L Villar Alvarez F Varela Crespo C Moreno Gomez Limon I Criado Jimenez A. Thoracic epidural anesthesia decreases C-reactive protein levels in patients undergoing elective coronary artery bypass graft surgery with cardiopulmonary bypass. Minerva Anestesiol. (2008) 74:619–26. 18971890

[37] Tenenbein PK Debrouwere R Maguire D Duke PC Muirhead B Enns J Thoracic epidural analgesia improves pulmonary function in patients undergoing cardiac surgery. Can J Anaesth. (2008) 55:344–50. 10.1007/BF03021489 18566197

[38] Lenkutis T Benetis R Sirvinskas E Raliene L Judickaite L. Effects of epidural anesthesia on intrathoracic blood volume and extravascular lung water during on-pump cardiac surgery. Perfusion. (2009) 24:243–8. 10.1177/0267659109348724 19808745

[39] Mehta Y Vats M Sharma M Arora R Trehan N. Thoracic epidural analgesia for off-pump coronary artery bypass surgery in patients with chronic obstructive pulmonary disease. Ann Card Anaesth. (2010) 13:224–30. 10.4103/0971-9784.69062 20826963

[40] Sharma M Mehta Y Sawhney R Vats M Trehan N. Thoracic epidural analgesia in obese patients with body mass index of more than 30 kg/m^2^ for off pump coronary artery bypass surgery. Ann Card Anaesth. (2010) 13:28–33. 10.4103/0971-9784.58831 20075532

[41] Caputo M Alwair H Rogers CA Pike K Cohen A Monk C Thoracic epidural anesthesia improves early outcomes in patients undergoing off-pump coronary artery bypass surgery: a prospective, randomized, controlled trial. Anesthesiology. (2011) 114:380–90. 10.1097/ALN.0b013e318201f571 21245735

[42] Kirov MY Eremeev AV Smetkin AA Bjertnaes LJ. Epidural anesthesia and postoperative analgesia with ropivacaine and fentanyl in off-pump coronary artery bypass grafting: a randomized, controlled study. BMC Anesthesiol. (2011) 11:17. 10.1186/1471-2253-11-17 21923942 PMC3182129

[43] Svircevic V Nierich AP Moons KG Diephuis JC Ennema JJ Brandon Bravo Bruinsma GJ Thoracic epidural anesthesia for cardiac surgery: a randomized trial. Anesthesiology. (2011) 114:262–70. 10.1097/ALN.0b013e318201d2de 21239976

[44] El-Morsy GZ El-Deeb A. The outcome of thoracic epidural anesthesia in elderly patients undergoing coronary artery bypass graft surgery. Saudi J Anaesth. (2012) 6:16–21. 10.4103/1658-354X.93048 22412771 PMC3299108

[45] Nielsen DV Bhavsar R Greisen J Ryhammer PK Sloth E Jakobsen CJ. High thoracic epidural analgesia in cardiac surgery. Part 2–high thoracic epidural analgesia does not reduce time in or improve quality of recovery in the intensive care unit. J Cardiothorac Vasc Anesth. (2012) 26:1048–54. 10.1053/j.jvca.2012.05.008 22770692

[46] Gurses E Berk D Sungurtekin H Mete A Serin S. Effects of high thoracic epidural anesthesia on mixed venous oxygen saturation in coronary artery bypass grafting surgery. Med Sci Monit: Int Med J Exp Clin Res Med. (2013) 19:222–9. 10.12659/MSM.883861 PMC3628587 23531633

[47] Onan B Onan IS Kilickan L Sanisoglu I. Effects of epidural anesthesia on acute and chronic pain after coronary artery bypass grafting. J Card Surg. (2013) 28:248–53. 10.1111/jocs.12086 23461638

[48] Neuburger PJ Ngai JY Chacon MM Luria B Manrique-Espinel AM Kline RP A prospective randomized study of paravertebral blockade in patients undergoing robotic mitral valve repair. J Cardiothorac Vasc Anesth. (2015) 29:930–6. 10.1053/j.jvca.2014.10.010 25620765

[49] Zawar BP Mehta Y Juneja R Arora D Raizada A Trehan N. Nonanalgesic benefits of combined thoracic epidural analgesia with general anesthesia in high risk elderly off pump coronary artery bypass patients. Ann Card Anaesth. (2015) 18:385–91. 10.4103/0971-9784.159810 26139745 PMC4881722

[50] Doğan Bakı E Kavrut Ozturk N Ayoğlu RU Emmiler M Karslı B Uzel H. Effects of parasternal block on acute and chronic pain in patients undergoing coronary artery surgery. Semin Cardiothorac Vasc Anesth. (2016) 20:205–12. 10.1177/1089253215576756 25900900

[51] Ozturk NK Baki ED Kavakli AS Sahin AS Ayoglu RU Karaveli A Comparison of transcutaneous electrical nerve stimulation and parasternal block for postoperative pain management after cardiac surgery. Pain Res Manag. (2016) 2016:4261949. 10.1155/2016/4261949 27445610 PMC4904586

[52] Lockwood GG Cabreros L Banach D Punjabi PP. Continuous bilateral thoracic paravertebral blockade for analgesia after cardiac surgery: a randomised, controlled trial. Perfusion. (2017) 32:591–7. 10.1177/0267659117715507 28592166

[53] Zhan Y Chen G Huang J Hou B Liu W Chen S. Effect of intercostal nerve block combined with general anesthesia on the stress response in patients undergoing minimally invasive mitral valve surgery. Exp Ther Med. (2017) 14:3259–64. 10.3892/etm.2017.4868 28912876 PMC5585755

[54] Kumar KN Kalyane RN Singh NG Nagaraja PS Krishna M Babu B Efficacy of bilateral pectoralis nerve block for ultrafast tracking and postoperative pain management in cardiac surgery. Ann Card Anaesth. (2018) 21:333–8. 10.4103/aca.ACA_15_18 30052231 PMC6078028

[55] Nagaraja PS Ragavendran S Singh NG Asai O Bhavya G Manjunath N Comparison of continuous thoracic epidural analgesia with bilateral erector spinae plane block for perioperative pain management in cardiac surgery. Ann Card Anaesth. (2018) 21:323–7. 10.4103/aca.ACA_16_18 30052229 PMC6078032

[56] Obersztyn M Trejnowska E Nadziakiewicz P Knapik P. Evaluation of thoracic epidural analgesia in patients undergoing coronary artery bypass surgery - a prospective randomized trial. Kardiochir TorakoChirurgia Pol. (2018) 15:72–8. 10.5114/kitp.2018.76471 30069186 PMC6066681

[57] Venkataswamy M Ramakrishna PS Nagaraja PS Singh NG Adoni PJ. Efficacy of bilateral continuous paravertebral block for off pump coronary artery bypass surgery. J Cardiovasc Dis Res. (2018) 9:59–62. 10.5530/jcdr.2018.2.15

[58] Fujii S Roche M Jones PM Vissa D Bainbridge D Zhou JR. Transversus thoracis muscle plane block in cardiac surgery: a pilot feasibility study. Reg Anesth Pain Med. (2019) 44:556–60. 10.1136/rapm-2018-100178 30902911

[59] Krishna SN Chauhan S Bhoi D Kaushal B Hasija S Sangdup T Bilateral erector spinae plane block for acute post-surgical pain in adult cardiac surgical patients: a randomized controlled trial. J Cardiothorac Vasc Anesth. (2019) 33:368–75. 10.1053/j.jvca.2018.05.050 30055991

[60] Lee CY Robinson DA Johnson CA Zhang Y Wong J Joshi DJ A randomized controlled trial of liposomal bupivacaine parasternal intercostal block for sternotomy. Ann Thorac Surg. (2019) 107:128–34. 10.1016/j.athoracsur.2018.06.081 30170012

[61] Sun L Li Q Wang Q Ma F Han W Wang M. Bilateral thoracic paravertebral block combined with general anesthesia vs. General anesthesia for patients undergoing off-pump coronary artery bypass grafting: a feasibility study. BMC Anesthesiol. (2019) 19:101. 10.1186/s12871-019-0768-9 31185919 PMC6560727

[62] Aydin ME Ahiskalioglu A Ates I Tor IH Borulu F Erguney OD Efficacy of ultrasound-guided transversus thoracic muscle plane block on postoperative opioid consumption after cardiac surgery: a prospective, randomized, double-blind study. J Cardiothorac Vasc Anesth. (2020) 34:2996–3003. 10.1053/j.jvca.2020.06.044 32665179

[63] El Shora HA El Beleehy AA Abdelwahab AA Ali GA Omran TE Hassan EA Bilateral paravertebral block versus thoracic epidural analgesia for pain control post-cardiac surgery: a randomized controlled trial. Thorac Cardiovasc Surg. (2020) 68:410–6. 10.1055/s-0038-1668496 30114712

[64] Gautam S Pande S Agarwal A Agarwal SK Rastogi A Shamshery C Evaluation of serratus anterior plane block for pain relief in patients undergoing MIDCAB surgery. Innov: Technol Tech Cardiothorac Vasc Surg. (2020) 15:148–54. 10.1177/1556984520908962 32352903

[65] Magoon R Kaushal B Chauhan S Bhoi D Bisoi A Khan M. A randomised controlled comparison of serratus anterior plane, pectoral nerves and intercostal nerve block for post-thoracotomy analgesia in adult cardiac surgery. Indian J Anaesth. (2020) 64:1018–24. 10.4103/ija.IJA_327_20 33542564 PMC7852449

[66] Vilvanathan S Saravanababu MS Sreedhar R Gadhinglajkar SV Dash PK Sukesan S. Ultrasound-guided modified parasternal intercostal nerve block: role of preemptive analgesic adjunct for mitigating poststernotomy pain. Anesth Essays Res. (2020) 14:300–4. 10.4103/aer.AER_32_20 33487833 PMC7819423

[67] Athar M Parveen S Yadav M Siddiqui OA Nasreen F Ali S A randomized double-blind controlled trial to assess the efficacy of ultrasound-guided erector spinae plane block in cardiac surgery. J Cardiothorac Vasc Anesth. (2021) 35:3574–80. 10.1053/j.jvca.2021.03.009 33832806

[68] Bloc S Perot BP Gibert H Law Koune JD Burg Y Leclerc D Efficacy of parasternal block to decrease intraoperative opioid use in coronary artery bypass surgery via sternotomy: a randomized controlled trial. Reg Anesth Pain Med. (2021) 46:671–8. 10.1136/rapm-2020-102207 33990437

[69] Khera T Murugappan KR Leibowitz A Bareli N Shankar P Gilleland S Ultrasound-guided pecto-intercostal fascial block for postoperative pain management in cardiac surgery: a prospective, randomized, placebo-controlled trial. J Cardiothorac Vasc Anesth. (2021) 35:896–903. 10.1053/j.jvca.2020.07.058 32798172

[70] Kumar AK Chauhan S Bhoi D Kaushal B. Pectointercostal fascial block (PIFB) as a novel technique for postoperative pain management in patients undergoing cardiac surgery. J Cardiothorac Vasc Anesth. (2021) 35:116–22. 10.1053/j.jvca.2020.07.074 32859487

[71] Wasfy SF Kamhawy GA Omar AH Abd El Aziz HF. Bilateral continuous erector spinae block versus multimodal intravenous analgesia in coronary bypass surgery. A randomized trial. Egypt J Anaesth. (2021) 37:152–8. 10.1080/11101849.2021.1904548

[72] Zhang Y Gong H Zhan B Chen S. Effects of bilateral pecto-intercostal fascial block for perioperative pain management in patients undergoing open cardiac surgery: a prospective randomized study. BMC Anesthesiol. (2021) 21:175. 10.1186/s12871-021-01391-w 34157970 PMC8218527

[73] Zhang Y Li X Chen S. Bilateral transversus thoracis muscle plane block provides effective analgesia and enhances recovery after open cardiac surgery. J Card Surg. (2021) 36:2818–23. 10.1111/jocs.15666 34047403

[74] Bjornnes AK Rustoen T Lie I Watt-Watson J Leegaard M. Pain characteristics and analgesic intake before and following cardiac surgery. Eur J Cardiovasc Nurs. (2016) 15:47–54. 10.1177/1474515114550441 25192967

[75] Zubrzycki M Liebold A Skrabal C Reinelt H Ziegler M Perdas E Assessment and pathophysiology of pain in cardiac surgery. J Pain Res. (2018) 11:1599–611. 10.2147/JPR.S162067 30197534 PMC6112778

[76] McCoul ED Barnett ML Brenner MJ. Reducing opioid prescribing and consumption after surgery-keeping the lock on pandora’s box. JAMA Otolaryngol Head Neck Surg. (2021) 147:819–21. 10.1001/jamaoto.2021.1846 34351391

[77] Clowes GH Jr Neville WE Hopkins A Anzola J Simeone FA. Factors contributing to success or failure in the use of a pump oxygenator for complete by-pass of the heart and lung, experimental and clinical. Surgery. (1954) 36:557–79. 13195976

[78] Guay J Kopp S. Epidural analgesia for adults undergoing cardiac surgery with or without cardiopulmonary bypass. Cochrane Database Syst Rev. (2019) 3:CD006715. 10.1002/14651858.CD006715.pub3 30821845 PMC6396869

[79] Zhang S Wu X Guo H Ma L. Thoracic epidural anesthesia improves outcomes in patients undergoing cardiac surgery: meta-analysis of randomized controlled trials. Eur J Med Res. (2015) 20:25. 10.1186/s40001-015-0091-y 25888937 PMC4375848

[80] Adler J Malone D. Early mobilization in the intensive care unit: a systematic review. Cardiopulm Phys Ther J. (2012) 23:5–13. 10.1097/01823246-201223010-00002 22807649 PMC3286494

[81] Sousa-Uva M Head SJ Milojevic M Collet JP Landoni G Castella M 2017 EACTS guidelines on perioperative medication in adult cardiac surgery. Eur J Cardiothorac Surg. (2018) 53:5–33. 10.1093/ejcts/ezx314 29029110

[82] Amat-Santos IJ Dumont E Villeneuve J Doyle D Rheault M Lavigne D Effect of thoracic epidural analgesia on clinical outcomes following transapical transcatheter aortic valve implantation. Heart. (2012) 98:1583–90. 10.1136/heartjnl-2012-302185 22791654

[83] Landoni G Isella F Greco M Zangrillo A Royse CF. Benefits and risks of epidural analgesia in cardiac surgery. Br J Anaesth. (2015) 115:25–32. 10.1093/bja/aev201 26089444

[84] Bracco D Hemmerling T. Epidural analgesia in cardiac surgery: an updated risk assessment. Heart Surg Forum. (2007) 10:E334–7. 10.1532/HSF98.20071077 17599887

[85] Liu H Emelife PI Prabhakar A Moll V Kendrick JB Parr AT Regional anesthesia considerations for cardiac surgery. Best Pract Res Clin Anaesthesiol. (2019) 33:387–406. 10.1016/j.bpa.2019.07.008 31791558

[86] Mace L. An audit of post-operative nausea and vomiting, following cardiac surgery: scope of the problem. Nurs Crit Care. (2003) 8:187–96. 10.1046/j.1362-1017.2003.00029.x 14653525

[87] Allen KB Brovman EY Chhatriwalla AK Greco KJ Rao N Kumar A Opioid-related adverse events: incidence and impact in patients undergoing cardiac surgery. Semin Cardiothorac Vasc Anesth. (2020) 24:219–26. 10.1177/1089253219888658 31771422

[88] Yeung JH Gates S Naidu BV Wilson MJ Gao Smith F. Paravertebral block versus thoracic epidural for patients undergoing thoracotomy. Cochrane Database Syst Rev. (2016) 2:CD009121. 10.1002/14651858.CD009121.pub2 26897642 PMC7151756

[89] Scarfe AJ Schuhmann-Hingel S Duncan JK Ma N Atukorale YN Cameron AL. Continuous paravertebral block for post-cardiothoracic surgery analgesia: a systematic review and meta-analysis. Eur J Cardiothorac Surg. (2016) 50:1010–8. 10.1093/ejcts/ezw168 27242357

[90] Forero M Adhikary SD Lopez H Tsui C Chin KJ. The erector spinae plane block: a novel analgesic technique in thoracic neuropathic pain. Reg Anesth Pain Med. (2016) 41:621–7. 10.1097/AAP.0000000000000451 27501016

[91] Balan C Bubenek-Turconi SI Tomescu DR Valeanu L. Ultrasound-guided regional anesthesia-current strategies for enhanced recovery after cardiac surgery. Medicina. (2021) 57:312. 10.3390/medicina57040312 33806175 PMC8065933

[92] Smith LM Barrington MJ, M. St Vincent's Hospital. Ultrasound-guided blocks for cardiovascular surgery: which block for which patient? Curr Opin Anaesthesiol. (2020) 33:64–70. 10.1097/ACO.0000000000000818 31833864

[93] de la Torre PA Garcia PD Alvarez SL Miguel FJ Perez MF. A novel ultrasound-guided block: a promising alternative for breast analgesia. Aesthet Surg J. (2014) 34:198–200. 10.1177/1090820X13515902 24396082

[94] Ueshima H Kitamura A. Blocking of multiple anterior branches of intercostal nerves (Th2-6) using a transversus thoracic muscle plane block. Reg Anesth Pain Med. (2015) 40:388. 10.1097/AAP.0000000000000245 26079353

[95] Piraccini E Calli M Corso RM Maitan S. Pectointercostal fascial block (PIFB) and parasternal block (PSB): two names for the same block? J Clin Anesth. (2019) 58:130. 10.1016/j.jclinane.2019.07.010 31377670

[96] Blanco R. The ‘pecs block’: a novel technique for providing analgesia after breast surgery. Anaesthesia. (2011) 66:847–8. 10.1111/j.1365-2044.2011.06838.x 21831090

[97] Yu S Valencia MB Roques V Aljure OD. Regional analgesia for minimally invasive cardiac surgery. J Card Surg. (2019) 34:1289–96. 10.1111/jocs.14177 31441548

[98] Albrecht E Chin KJ. Advances in regional anaesthesia and acute pain management: a narrative review. Anaesthesia. (2020) 75(Suppl 1):e101–10. 10.1111/anae.14868 31903582

