# Supplementary material for: Comparison of regional anesthetic techniques for postoperative analgesia after adult cardiac surgery: bayesian network meta-analysis
Source: Front Cardiovasc Med. 2023 May 22;10:1078756. doi: 10.3389/fcvm.2023.1078756 (PMC10239891; doi:10.3389/fcvm.2023.1078756)
Supplement: Supplementary file 1 [file Datasheet1.pdf]

Comparison of regional anesthetic techniques for postoperative analgesia after adult cardiac surgery: Bayesian network meta-analysis

Content

Table S1. Search strategies 2

Table S2. Characteristics of included studies 3

Table S3. Head-to-head comparisons of pain score 2-4h at rest 7

Table S4. Head-to-head comparisons of pain score 2-4h at cough 7

Table S5. Head-to-head comparisons of pain score 6h at rest 7

Table S6. Head-to-head comparisons of pain score 6h at cough 7

Table S7. Head-to-head comparisons of pain score 12h at rest 7

Table S8. Head-to-head comparisons of pain score 12h at cough 7

Table S9. Head-to-head comparisons of pain score 24h at rest 7

Table S10. Head-to-head comparisons of pain score 24h at cough 7

Table S11. Head-to-head comparisons of pain score 48h at rest 7

Table S12. Head-to-head comparisons of pain score 48h at cough 7

Table S13. Head-to-head comparisons of cumulative morphine consumption 24h 8

Table S14. Head-to-head comparisons of cumulative morphine consumption 48h 8

Table S15. Head-to-head comparisons of need for rescue analgesia 8

Table S16. Head-to-head comparisons of postoperative nausea and vomiting......8

Table S17. Head-to-head comparisons of pruritus 8

Table S18. Head-to-head comparisons of time to tracheal extubation......8

Table S19. Head-to-head comparisons of intensive care unit stay......8

Table S20. Head-to-head comparisons of hospital stay 8

Table S21. Head-to-head comparisons of mortality 8

Table S22. Assessment of publication bias 9

Table S23. Ongoing trials with regional anesthetic techniques for cardiac surgery 9

Figure S1. Risk of bias summary. 10

Figure S2. Risk of bias graph. 11

Figure S3. Network plot of eligible comparisons among different regional anesthetic techniques.12

Figure S4. Inconsistency test of pain scores 13

Figure S5. Inconsistency test of other outcomes 14

PRISMA Network Meta-analysis Checklist 15

e-Reference 16

Table S1. Search strategies

	Strategy
**PubMed**	#1: (randomized controlled trial[Publication Type] OR controlled clinical trial[Publication Type] OR randomized[Title/Abstract] OR random[Title/Abstract] OR randomly[Title/Abstract] OR controlled[Title/Abstract] OR trial[Title/Abstract] OR placebo[Title/Abstract] OR groups[Title/Abstract]) NOT ((animals [Mesh] OR swine [Title/Abstract] OR pig [Title/Abstract] OR pigs[Title/Abstract] OR piglet*[Title/Abstract] OR rat[Title/Abstract] OR mouse[Title/Abstract]) NOT humans [Mesh])<br><br>#2: Cardiac Surgical Procedures[Mesh] OR ((card*[Title/Abstract] OR heart[Title/Abstract] OR valve[Title/Abstract] OR aortic[Title/Abstract]) AND surg*[Title/Abstract]) OR coronary artery bypass grafting[Title/Abstract] OR coronary bypass[Title/Abstract] OR CABG[Title/Abstract] OR ventricular assist device[Title/Abstract] OR defibrillator[Title/Abstract] OR device closure[Title/Abstract] OR cardiopulmonary bypass[Title/Abstract]<br><br>#3: (thoracic epidural analgesia[Title/Abstract] OR thoracic epidural block*[Title/Abstract] OR TEA[Title/Abstract]) OR (paravertebral block*[Title/Abstract] OR PVB[Title/Abstract] OR TPVB[Title/Abstract]) OR (erector spinae plane block*[Title/Abstract] OR erector spinae block*[Title/Abstract] OR erector spinae plane[Title/Abstract] OR ESB[Title/Abstract] OR ESP[Title/Abstract] OR ESPB[Title/Abstract]) OR (serratus anterior plane block*[Title/Abstract] OR serratus anterior block*[Title/Abstract] OR serratus anterior plane[Title/Abstract] OR SAP block*[Title/Abstract] OR SAPB[Title/Abstract]) OR (pectoral nerve block*[Title/Abstract] OR pectoralis nerve block*[Title/Abstract] OR pectoral block*[Title/Abstract] OR pectoralis block*[Title/Abstract] OR PECS[Title/Abstract]) OR (transversus thoracic muscle plane block*[Title/Abstract] OR transversus thoracis muscle plane block*[Title/Abstract] OR TTPB[Title/Abstract] OR TTMPB[Title/Abstract] OR TTMP block*[Title/Abstract]) OR (pecto-intercostal fascial block*[Title/Abstract] OR pectointercostal fascia block*[Title/Abstract] OR PIF[Title/Abstract] OR PIFB[Title/Abstract] OR (parasternal block[Title/Abstract] OR intercostal nerve block*[Title/Abstract] OR interfascial*[Title/Abstract])<br><br>#4: #1 AND #2 AND #3
**MEDLINE**	#1: (randomized controlled trial.pt OR controlled clinical trial.pt OR randomized.mp. OR placebo.mp. OR randomly.mp. OR trial.ti. OR Clinical Trials as Topic/) NOT (animals.sh. NOT humans.sh.)<br><br>#2: Cardiac Surgical Procedures.sh. OR ((card* OR heart OR valve OR aortic) AND surg*).mp. OR (coronary artery bypass grafting OR coronary bypass OR CABG OR ventricular assist device OR defibrillator OR device closure OR cardiopulmonary bypass).mp.<br><br>#3: (thoracic epidural analgesia OR thoracic epidural block* OR TEA).mp. OR (paravertebral block* OR PVB OR TPVB).mp. OR (erector spinae plane block* OR erector spinae block* OR erector spinae plane OR ESB OR ESP OR ESPB).mp. OR (serratus anterior plane block* OR serratus anterior block* OR serratus anterior plane OR SAP block* OR SAPB).mp. OR (pectoral nerve block* OR pectoralis nerve block* OR pectoral block* OR pectoralis block* OR PECS).mp. OR (Transversus thoracic muscle plane block* OR Transversus thoracis muscle plane block* OR TTPB OR TTMPB OR TTMP block*).mp. OR (pecto-intercostal fascial block* OR pectointercostal fascia block* OR PIF OR PIFB OR parasternal block OR Intercostal nerve block* OR Interfascial*).mp.<br><br>#4: #1 AND #2 AND #3
**Embase**	#1: 'random*':ab,ti OR 'placebo':ab,ti OR 'double-blind':ab,ti OR 'controlled':ab,ti OR 'group':ab,ti OR 'trial':ab,ti<br><br>#2: 'heart surgery'/exp OR ((card*:ab,ti OR heart:ab,ti OR valve:ab,ti OR aortic:ab,ti) AND surg*:ab,ti) OR 'coronary artery bypass grafting':ab,ti OR 'coronary bypass':ab,ti OR 'CABG':ab,ti OR 'ventricular assist device':ab,ti OR 'defibrillator':ab,ti OR 'device closure':ab,ti OR 'cardiopulmonary bypass':ab,ti<br><br>#3: 'thoracic epidural analgesia':ab,ti OR 'thoracic epidural block*':ab,ti OR 'paravertebral block*':ab,ti OR 'erector spinae plane block*':ab,ti OR 'erector spinae block*':ab,ti OR 'erector spinae plane':ab,ti OR 'serratus anterior plane block*':ab,ti OR 'serratus anterior block*':ab,ti OR 'serratus anterior plane':ab,ti OR 'SAP block*':ab,ti OR 'pectoral nerve block*':ab,ti OR 'pectoralis nerve block*':ab,ti OR 'pectoral block*':ab,ti OR 'pectoralis block*':ab,ti OR 'PECS':ab,ti OR 'Transversus thoracic muscle plane block*':ab,ti OR 'Transversus thoracis muscle plane block*':ab,ti OR 'pecto-intercostal fascial block*':ab,ti OR 'pectointercostal fascia block*':ab,ti OR 'parasternal block':ab,ti OR 'Intercostal nerve block*':ab,ti OR 'Interfascial*':ab,ti<br><br>#4: #1 AND #2 AND #3

Table S2. Characteristics of included studies

Author	Year	Country	Surgery type	Group	Number	Regional anesthetics (Drug, dose)	Block timing	Postoperative analgesia	Outcomes
el-Baz[1]	1987	USA	CABG	TEA	30	1 mL/h of 0.1 mg/mL morphine sulfate	Post-operation	Morphine IV 2 mg/2h	②④⑥
				Control	30	No treatment			
Rein[2]	1989	Norway	CABG	TEA	8	Induced by 50 mg 0.5% bupivacaine, maintained with 20 mg/h	Pre-operation	Continued for 24h	⑨
				Control	8	No treatment		Morphine	
Leim[3]	1992	Netherlands	CABG	TEA	25	0.05 mL/cm/h of 0.125% bupivacaine and sufentanil	Pre-operation	Continued for 72h, morphine IV	①③⑥
				Control	25	No treatment		0.1 mg/kg/6h of nicomorphine IV	
Stenseth[4]	1996	Norway	CABG	TEA	26	10 mL of 5 mg/mL bupivacaine	Pre-operation	3 mL/h of 5 mg/mL bupivacaine, morphine	②⑥⑨
				Control	26	No treatment		Morphine IV for 20h, codeine, paracetamol	
Fawcett[5]	1997	UK	CABG	TEA	8	15 mL of 0.5% bupivacaine	Pre-operation	5–8 mL/h of 0.375% bupivacaine for 24h	①
				Control	8	No treatment		Morphine IV 40 μg/kg/h for 24h	
Brix-Christensen[6]	1998	Denmark	CABG	TEA	8	5 mL/h of 2 mg/mL bupivacaine and fentanyl 5 μg/mL	Post-operation	Continued until the POD-2	⑥
				Control	8	No treatment		Morphine, paracetamol	
Loick[7]	1999	Germany	CABG	TEA	25	8–12 mL of 0.375% bupivacaine and 16–24 μg of sufentanil	Pre-operation	2–3 mL of 0.75% bupivacaine, 1 mg/mL sufentanil	①②
				Control	21	No treatment		Piritramide PCA, 1 g of paracetamol QID	
Tenling[8]	1999	Sweden	CABG	TEA	14	4–8 mL/h of 5 mg/mL bupivacaine	Pre-operation	Continued with 1 μg/mL sufentanil, ketobemidone IV, meperidine	⑥
				Control	14	No treatment		Ketobemidone IV, meperidine	
Dhole[9]	2001	India	CABG	TEA	20	Induced by 8 mL of 0.5% bupivacaine, maintained with 6 mL/h of 0.25% bupivacaine	Pre-operation	Continued until moving to the recovery room, ketorolac trimethamine IM	①③⑥
				PVB	20				
Jidéus[10]	2001	Sweden	CABG	TEA	41	Induced by 8–14 mL of 5 mg/mL bupivacaine, maintained with 4–8 mL/h	Pre-operation	2 mg/mL bupivacaine, 3–7 mL/h of 1 μg/ml sufentanil	⑥
				Control	80	No treatment		Ketobemidone	
Scott[11]	2001	UK	CABG	TEA	206	Induced by 10 mL of 0.5% bupivacaine, maintained with 10 mL/h of 0.125% bupivacaine and 0.0006% clonidine (300 g in 500 mL)	Pre-operation	Continued for 96h, oral ibuprofen and co-proxamol	⑨
				Control	202	No treatment		Alfentanil for 24h, followed by morphine PCA for 48h; oral ibuprofen and co-proxamol	
Bach[12]	2002	Germany	CABG	TEA	13	Induced by 10 mL of 0.25% bupivacaine, maintained with (Height-100) mL/h/cm of 0.25% bupivacaine	Pre-operation	Continued until POD-2 or POD-3	⑨
				Control	15	Saline IV		NR	
de Vries[13]	2002	Netherlands	CABG	TEA	28	8–10 mL of 0.25% bupivacaine and 2.5 μg/mL sufentanil	Pre-operation	8–10 mL/h of 0.125% bupivacaine and 0.5 μg/mL sufentanil, paracetamol	①⑧
				Control	57	No treatment		Piritramide IM 0.2 mg/kg, paracetamol	
Fillinger[14]	2002	USA	CABG	TEA	30	Induced by 20 g/kg morphine and 25–35 mg 0.5% bupivacaine, maintained with 4–10 mL/h of 0.5% bupivacaine and 25 μg/mL morphine	Pre-operation	4–10 mL/h of 0.125% bupivacaine, 25 μg/mL morphine until POD-1	①②⑥⑦⑧
				Control	30	No treatment		Morphine IV, oral hydrocodone	
Priestley[15]	2002	Australia	CABG	TEA	50	Induced by 4 mL ropivacaine 1% and fentanyl 100 μg, maintained with 3–5 mL/h of 1% ropivacaine, 5 μg/mL fentanyl	Pre-operation	Continued for 48h, followed by meperidine PCA for 24h; naproxen, paracetamol, oxycodone/codeine	①③⑥⑧
				Control	50	No treatment		Morphine PCA for 72h, naproxen, paracetamol, oxycodone/codeine	
Berendes[16]	2003	Germany	CABG	TEA	36	Induced by 6–12 mL of 0.5% bupivacaine hydrochloride and 15–25 μg of sufentanil citrate, maintained with 2 mL/h of 0.75% bupivacaine hydrochloride	Pre-operation	Continued using PCA	⑥⑨
				Control	37	No treatment		NR	
Royse[17]	2003	Australia	CABG	TEA	37	Induced by 8 mL of 0.5% ropivacaine with 20 μg of fentanyl, maintained with 5–14 mL of 0.2% ropivacaine and 2 μg/mL of fentanyl	Pre-operation	Continued until POD-3 6:00 AM, acetaminophen/indomethacin/ tramadol	①⑦⑧⑨
				Control	39	No treatment		Morphine PCA until POD-3 6:00 AM, acetaminophen/indomethacin/ tramadol	
Volk[18]	2003	Germany	CABG	TEA	13	6–10 mL of 0.5% bupivacaine	Pre-operation	6–12 mL/h of 0.25% bupivacaine for 24h, piritramide	①③⑦⑧
				Control	13	No treatment		Piritramide PCA	
Kendall[19]	2004	UK	CABG	TEA	10	Induced by 0.1 mL/kg of 0.1% bupivacaine and 5 μg/mL fentanyl, maintained with the rate of 0.1 mL/kg/h	Pre-operation	Continued for 48h, paracetamol, codeine	⑨
				Control	20	No treatment		0.1 mg/kg Morphine PCA, paracetamol, codeine	
Nygård[20]	2004	Denmark	CABG	TEA	44	5 mL/h of 1.25 mg/mL bupivacaine and 25 μg/mL morphine; additional bolus doses of 4 mL/h of 5 mg/mL bupivacaine during the operation	Pre-operation	Continued for 4 days	⑥⑧
				Control	48	No treatment		2.5–5 mg morphine IV until POD-1 morning; 5–10 mg oral morphine	

Table S2. Continue

Author	Year	Country	Surgery type	Group	Number	Regional anesthetics (Drug, dose)	Block timing	Postoperative analgesia	Outcomes
Barrington[21]	2005	Australia	CABG	TEA	60	Induced by 5 mL of 1% ropivacaine and 50 µg fentanyl, maintained with 0.2% ropivacaine and 12 µg/mL fentanyl	Pre-operation	Continued until POD-3 morning, acetaminophen, indomethacin, oxycodone	①③⑥⑧⑨
				Control	60	No treatment		0.1–0.2 mg/kg morphine, 0.3 mg/kg ropivacaine until POD-2 morning, acetaminophen, indomethacin, oxycodone	
McDonald[22]	2005	USA	Mixed	PINB	8	54 mL of 0.25% levobupivacaine: 2 mL for each of the 5 interspaces bilaterally (total of 20 mL); 12 mL per side (total of 24 mL) just above the periosteum along the lateral borders of the sternum; 10 mL deeply and evenly around the mediastinal tubes	Post-operation	Morphine IV 2 mg for 8h, ketorolac IV 15–30 mg	①②③
				Control	9	No treatment			
Hansdottir[23]	2006	Sweden	CABG	TEA	53	Induced by 0.1 mL/kg of bupivacaine, maintained with 5 mL/kg/h of 5 mg/mL bupivacaine	Pre-operation	0.1 mL/kg/h of 1 mg/mL bupivacaine with 2 µg/mL fentanyl until POD-4 morning, oral acetaminophen	①⑥⑧
				Control	55	No treatment		Morphine PCA 0.01 mg/kg until POD-4 morning, oral acetaminophen	
Bakhtiary[24]	2007	Germany	CABG	TEA	66	2–5 mL/h of 0.16% ropivacaine and 1 µg/mL sufentanil	Pre-operation	Continued for 3 days	⑥
				Control	66	No treatment		NR	
Barr[25]	2007	Australia	Mixed	PINB	45	4 mL aliquots injected into the 5 anterior intercostal spaces T2-T6 (each side receiving 20 mL), with a total dosage of 40 mL (300 mg)	Pre-operation	Morphine/meperidine PCA 1 mg/mL for 24h, paracetamol, 50–100 mg tramadol IV, oral codeine	①②⑥
				Control	43	40 mL of 0.9% saline			
Kiliçkan[26]	2008	Turkey	CABG	TEA	15	20 mg/h of 0.25% bupivacaine	Pre-operation	Continued for 3 days	⑥⑧
				Control	15	No treatment		Dolantin PCA	
Mehta[27]	2008	India	CABG	TEA	19	Induced by 8 mL of 0.5% bupivacaine, maintained with 0.1 mL/kg/h of 0.25% bupivacaine	Pre-operation	Continued, diclofenac sodium 75 mg IM	①⑥⑧
				PVB	17				
Palomero Rodríguez[28]	2008	Spain	CABG	TEA	10	Induced by 6–8 mL 0.33% bupivacaine, maintained with 6–8 mL/h of 0.175% bupivacaine	Pre-operation	Continued for 48h	⑥⑨
				Control	12	No treatment		0.5–1 mL/h morphine infusion	
Tenenbein[29]	2008	Canada	CABG	TEA	25	Induced by 5 mL of 0.75% ropivacaine and 200 µg of hydromorphone, maintained with 5 mL/h of 0.75% ropivacaine	Pre-operation	0.2% ropivacaine and 15 µg/mL hydromorphone for 48h, indomethacin, naproxen	①⑥
				Control	25	No treatment		Morphine PCA for 48h, indomethacin, naproxen	
Lenkutis[30]	2009	Lithuania	CABG	TEA	30	8 mL/h of 0.25% bupivacaine	Pre-operation	5–7 mL/h of 0.25% bupivacaine with 5 µg/mL fentanyl for 84h, pethidinum IM	⑥
				Control	30	No treatment		Pethidinum IV 0.1–0.4 mg/kg	
Mehta[31]	2010	India	CABG	TEA	31	Induced by 8–10 mL of 0.25% bupivacaine, maintained with 5 mL/h of 0.125% bupivacaine with 1 µg/mL fentanyl citrate	Pre-operation	Continued until POD-3	①③④⑤⑥
				Control	31	No treatment		Tramadol IV 1–2 mg/kg every eight hours	
Sharma[32]	2010	India	CABG	TEA	30	Induced by 8–10 mL of 0.25% bupivacaine, maintained with 5 mL/h of 0.125% bupivacaine with 1 µg/mL fentanyl citrate	Pre-operation	Continued until POD-3	①⑥⑦
				Control	30	No treatment		Tramadol hydrochloride IV 1–2 mg/kg	
Caputo[33]	2011	UK-Italy	CABG	TEA	109	Induced by 10 mL of 0.5% bupivacaine, maintained with 10 mL/h of 0.125% bupivacaine and 0.0003% clonidine	Pre-operation	Continued for 72h, oral paracetamol	⑥⑦⑧⑨
				Control	117	No treatment		Morphine PCA for 48h, oral paracetamol	
Kirov[34]	2011	Russian	CABG	TEA	60	1 mg/kg of 0.75% ropivacaine, 1 µg/mL fentanyl	Pre-operation	3–8 mL/h of 0.2% ropivacaine, 2 µg/mL fentanyl, lornoxicam 8 mg IV every 12h for two days	①⑥⑦⑧
				Control	30	No treatment		3–8 mL/h of 10 µg/mL fentanyl IV, lornoxicam 8 mg IV every 12h for two days	
Svircevic[35]	2011	Netherlands	Mixed	TEA	325	Induced by 0.1 mL/kg of 0.08 mg/mL morphine and 0.125 mg/mL bupivacaine, maintained with the rate of 4–8 mL/h	Pre-operation	Continued until discharge from the ICU, followed with morphine, oral paracetamol	①⑥⑦⑧⑨
				Control	329	No treatment		1–4 mg/h morphine, oral paracetamol	
El-Morsy[36]	2012	Egypt	CABG	TEA	25	5 mL/h of 0.125 mg/mL bupivacaine, 1 µg/mL fentanyl	Pre-operation	Continued with 1–2 mg/kg tramadol IV for 24h	①④⑤⑥⑦⑧
				Control	25	No treatment		1–2 mg/kg tramadol IV for 24h	
Nielsen[37]	2012	Denmark	Mixed	TEA	30	Induced by 5–7 mL of 0.5% bupivacaine and 2.5 µg/mL sufentanil, maintained with 4–6 mL/h of 2.5 mg/mL bupivacaine and 1 µg/mL sufentanil	Pre-operation	Continued until POD-2 or POD-3, morphine IV or alfentanil, paracetamol	⑥⑨
				Control	30	No treatment		Morphine IV or alfentanil, paracetamol	

Table S2. Continue

Author	Year	Country	Surgery type	Group	Number	Regional anesthetics (Drug, dose)	Block timing	Postoperative analgesia	Outcomes
Gurses[38]	2013	Turkey	CABG	TEA	32	Induced by 10 mL of 0.075 mg/kg levobupivacaine hydroclorur and 2 μg/kg fentanyl, maintained with 0.0375 mg/kg/h of levobupivacaine, 1 μg/mL fentanyl	Pre-operation	Continued during ICU	③⑥⑦⑧
				Control	32	No treatment		Diclofenac sodium IM	
Onan[39]	2013	Turkey	CABG	TEA	20	Induced by 20 mg of 0.25% bupivacaine, maintained with the rate of 20 mg/h	Pre-operation	10–20 mL/h of 0.25% bupivacaine until discharge from the ICU, acetaminophen or tramadol	①②③⑥⑦⑧
				Control	20	No treatment		Acetaminophen or tramadol	
Neuburger[40]	2015	USA	Valve surgery	PVB	27	30 mL of 0.5% bupivacaine into 4 right-sided injections from T3–T6	Pre-operation	PCA fentanyl for 24h	①⑧
				Control	23	No treatment			
Zawar[41]	2015	India	CABG	TEA	35	Induced by 6–14 mL ropivacaine 0.75%, maintained with 5–15 mL/h of 0.2% ropivacaine	Pre-operation	Continued for 72h	⑥⑦
				Control	46	No treatment		Tramadol hydrochloride IV every eight hours	
Doğan Bakı[42]	2016	Turkey	CABG	PINB	40	50 mL of study solution (levobupivacaine 25 mL, fentanyl 100 μg, 23 mL saline): bilateral 5 costa levels and every level 2 mL on both sides of the sternum, over sternal periosteum 20 mL and the entrance of chest tubes deep infiltration 10 mL	Post-operation	Tramadol PCA, paracetamol, Tenoxicam	②③⑥⑦
				Control	41	No treatment			
Ozturk[43]	2016	Turkey	Mixed	PINB	38	50 mL of study solution (25 mL levobupivacaine and 25 mL saline): 2 mL aliquots injected into five anterior (T2-T6) intercostal spaces on each side of the sternum and 20 mL over the periosteum, deeply infiltrated with 10 mL	Post-operation	Morphine PCA, acetaminophen, tramadol IV 50 mg	②⑥⑦⑧
				Control	37	Saline			
Lockwood[44]	2017	UK	CABG	PVB	25	20–30 mL of 0.5% plain lidocaine on each side (T3-T4)	Pre-operation	Morphine PCA, paracetamol	②⑥⑧
				Control	25	Sham catheters			
Zhan[45]*	2017	China	Valve surgery	PINB	15	3–5 mL of 0.5% ropivacaine (on each intercostal space (T3-T7)	Pre-operation	NR	①⑥⑦
				Control	15	No treatment			
Kumar[46]	2018	India	Mixed	PECS	20	30 mL of 0.25% bupivacaine with 25 μg dexmedetomidine on each side	Pre-operation	Paracetamol and tramadol IV	①③⑥
				Control	20	No treatment			
Nagaraja[47]	2018	India	Mixed	TEA	25	Induced by 15 mL of 0.25% bupivacaine, maintained with 0.1 mL/kg/h 0.125% plain bupivacaine	Pre-operation	Continued for 48h	①⑥⑦
				ESPB	25	Induced by 15 mL of 0.25% bupivacaine, maintained with 0.1 mL/kg/h 0.125% plain bupivacaine for each catheter			
Oberszytn[48]	2018	Poland	CABG	TEA	18	Induced by 12 mL of 0.25% bupivacaine with fentanyl in a concentration of 10 μg/mL, maintained with 4 mL/h of 40 mL 0.19% bupivacaine and fentanyl	Pre-operation	2–8 mL/h of 40 mL 0.125% bupivacaine and fentanyl (6.25 μg/mL), morphine PCA	⑥⑦⑧
				Control	18	No treatment		Morphine PCA	
Venkataswamy[49]	2018	India	CABG	PVB	30	Induced by 0.25 mL/kg of 0.25%Bupivacaine, maintained with 0.15 mL/kg/h of 0.125% bupivacaine for each catheter	Pre-operation	Continued, diclofenac 75mg IM	④⑥⑦⑧
				Control	30	No treatment		Tramadol IV 1mg/kg, diclofenac 75mg IM	
Fujii[50]	2019	Canada	Mixed	TTMPB	9	20 mL of 0.3% or 0.5% ropivacaine for each side	Post-operation	Hydromorphone, acetaminophen	①②⑥
				Control	8	Sham catheters			
Krishna[51]	2019	India	CABG	ESPB	53	20–25 mL of 3 mg/kg of 0.375% ropivacaine for each side	Pre-operation	NR	①⑥⑦
				Control	53	No treatment		Intravenous paracetamol, tramadol	
Lee[52]	2019	USA	CABG	PINB	38	50 mL of 0.53% Exparel: 4-mL injections per intercostal space across levels two through six bilaterally, followed by 10 mL infiltrated in the subcutaneous tissue surrounding mediastinal drains	Post-operation	Fentanyl/hydromorphone PCA, oral oxycodone-acetaminophen/hydrocodone-acetaminophen, ketorolac/tramadol	③
				Control	41	Saline			
Sun[53]	2019	China	CABG	PVB	29	Induced by 20 mL of 0.375% of ropivacaine twice (5 ml + 15 ml), maintained with 5 mL/h of 0.375% of ropivacaine for each side	Pre-operation	Morphine PCA 1 mg/mL for 48h	①②③④⑥⑦⑧
				Control	30	No treatment			
Aydin[54]	2020	Turkey	Mixed	TTMPB	24	20 mL of 0.25% bupivacaine for each side	Pre-operation	Fentanyl PCA, paracetamol, tramadol	①②③④⑤⑥⑦
				Control	24	Saline			

Table S2. Continue

Author	Year	Country	Surgery type	Group	Number	Regional anesthetics (Drug, dose)	Block timing	Postoperative analgesia	Outcomes
EI Shora[55]	2020	Egypt	Mixed	PVB	70	Induced by 6 mL of 0.125% bupivacaine and 1μ/mL fentanyl, maintained with the rate of 6 mL/h	Post-operation	Continued for 48h, morphine	①③④⑥⑦⑧⑨
				TEA	75				
Gautam[56]	2020	India	CABG	SAPB	25	8 mL/h of 20 ml (0.2% ropivacaine and1 μ/mL fentanyl)	Post-operation	Acetaminophen IV 1 g every six hours, fentanyl PCA	①②③④
				Control	25	8 mL/h of 20 ml saline			
Magoon[57*]	2020	India	Mixed	SAPB	30	2.5 mg/kg of 0.5% ropivacaine	Post-operation	Paracetamol IV, 15 mg/kg every eight hours, 0.5–1 μg/kg fentanyl IV	①⑥⑦
				PECS	31				
				PINB	30				
Vilvanathan[58]	2020	India	CABG	PINB	45	1.5 mL of 0.5% levobupivacaine was injected below the internal intercostal muscles to block the terminal anterior branch of the intercostal nerve (T2-T6) for each side. 3 mL of 0.5% levobupivacaine was infiltrated to block the superficial sensory nerves from T1 and supraclavicular nerve.	Pre-operation	Morphine at 20 μg/kg/h	③④⑤⑥
				Control	45	Saline			
Athar[59]	2021	India	Mixed	ESPB	15	20 mL of 0.25% levobupivacaine for each side	Pre-operation	Tramadol, paracetamol, fentanyl	①②④⑤⑥
				Control	15	Saline			
Bloc[60]	2021	France	CABG	PINB	18	60 mL of 0.25% ropivacaine divided into four injections of 15 mL (two per side, between ribs 2 and 3 and between ribs 4 and 5). The total amount of ropivacaine injected was therefore 150 mg.	Pre-operation	NR	⑥
				Control	17	Saline			
Khera[61]	2021	USA	Mixed	PIFB	40	20 mL of 0.25% bupivacaine for each side	Post-operation	Twice in ICU	②③⑦⑧⑨
				Control	40	Saline		NR	
Kumar[62]	2021	India	Mixed	PIFB	20	10 mL of 0.25% ropivacaine for each side	Post-operation	Paracetamol IV, tramadol, fentanyl IV	①⑥
				Control	20	No treatment			
Wasfy[63]	2021	Egypt	CABG	ESPB	20	15 mL of 0.25% bupivacaine for each side	Pre-operation	8 ml/h of 0.125%bupivacaine for 48h, morphine PCA	①②⑥⑦
				Control	20	No treatment		acetaminophen, ketorolac, morphine PCA	
Zhang[64]	2021	China	Valve surgery	PIFB	49	20 mL of 0.4% ropivacaine was injected to this plane in two locations, over 2nd and 4th rib for each side	Pre-operation	Sufentanil PCA, parecoxib IV	②④⑥⑦⑧
				Control	49	Saline			
Zhang[65]	2021	China	Mixed	TTMPB	30	20 mL of 0.4% ropivacaine for each side	Pre-operation	Sufentanil, flurbiprofen axetil	②⑥⑦⑧
				Control	30	Saline		flurbiprofen axetil	

CABG, coronary artery bypass grafting; ESPB, erector spinae plane block; IM indicates intramuscular; IV indicates intravenous; NR, not reported; PCA, patient-controlled analgesia; PECS, pectoral nerve block; PIFB, pecto-intercostal fascial block; PINB, parasternal intercostal nerve block; POD, postoperative day; PONV, postoperative nausea and vomiting; PVB, paravertebral block; SAPB, serratus anterior plane block; TEA, thoracic epidural analgesia; TTMPB, transversus thoracic muscle plane block. *: non-parasternal intercostal nerve block. Outcomes: ①Pain score; ②Cumulative IV morphine-equivalent consumption (mg); ③Need for rescue analgesia; ④PONV; ⑤Pruritus; ⑥Time to tracheal extubation (minutes); ⑦ICU stay (hours); ⑧Hospital stay (days); ⑨Mortality

Table S3. Head-to-head comparisons of pain score 2-4h at rest

Control	-11.57 (-32, 8.8)	-22.99 (-54.97, 9.83)	-10 (-48.97, 29.15)	-16.27 (-50.03, 18.62)	-21.74 (-53.03, 9.37)	-21.13 (-65.37, 23.89)	-18.54 (-40.99, 3.61)	-20 (-59.7, 19.72)
	ESPB	-11.39 (-49.63, 27.41)	1.61 (-42.6, 45.7)	-4.66 (-44.13, 35.91)	-10.16 (-45.82, 25.27)	-9.54 (-58.37, 40.01)	-6.99 (-32.81, 18.61)	-8.45 (-53.14, 36.23)
		PECS	12.93 (-38.1, 63.7)	6.69 (-25.66, 39.05)	1.22 (-44.15, 45.88)	1.86 (-35.54, 39.27)	4.37 (-35.24, 43.23)	2.96 (-48.65, 54.22)
			PIFB	-6.27 (-58.11, 46.27)	-11.73 (-62.17, 38.01)	-11.18 (-70.11, 48.34)	-8.58 (-53.96, 36.21)	-10.07 (-65.82, 45.93)
				PINB	-5.51 (-52.58, 40.4)	-4.82 (-42.6, 32.31)	-2.32 (-43.74, 37.97)	-3.69 (-56.66, 48.34)
					PVB	0.64 (-53.58, 55.45)	3.18 (-28.15, 34.45)	1.79 (-48.73, 52.34)
						SAPB	2.54 (-47.85, 51.91)	1.05 (-59.07, 60.7)
							TEA	-1.4 (-46.73, 44.17)
								TTMPB

Table S4. Head-to-head comparisons of pain score 2-4h at cough

Control	-35.14 (-100.44, 29.94)	-28.54 (-74.19, 17.5)	-19.99 (-65.39, 25.31)	-7 (-57.16, 43.82)	-33.22 (-99.14, 33.76)	-35.94 (-82.66, 10.69)	-29.84 (-75.99, 16.11)
	ESPB	6.58 (-73.62, 86.43)	15.08 (-64.1, 94.22)	28.1 (-53.83, 110.32)	2.05 (-63.13, 67.45)	-0.8 (-46.26, 44.52)	5.32 (-74.78, 85.02)
		PECS	8.51 (-55.96, 72.71)	21.51 (-46.26, 89.7)	-4.52 (-85.35, 76.36)	-7.41 (-72.86, 58.03)	-1.3 (-66.38, 63.92)
			PIFB	13.02 (-54.47, 81.48)	-13.08 (-92.89, 67.23)	-15.94 (-80.85, 49.12)	-9.84 (-74.56, 55.17)
				PINB	-26.09 (-108.95, 57.39)	-28.91 (-97.57, 39.47)	-22.92 (-91.03, 45.26)
					PVB	-2.86 (-49.96, 43.83)	3.28 (-77.96, 83.25)
						TEA	6.07 (-59.57, 71.66)
							TTMPB

Table S5. Head-to-head comparisons of pain score 6h at rest

Control	**-10.7 (-19.95, -2.24)**	**-16.14 (-33.13, -0.59)**	-10 (-26.63, 6.51)	-10.05 (-26.53, 6.9)	-11.01 (-26.07, 3.56)	-11.82 (-27.09, 3.71)	**-9.9 (-17.11, -3.12)**	**-20.01 (-37.48, -2.5)**
	ESPB	-5.34 (-24.43, 12.53)	0.67 (-17.84, 19.88)	0.71 (-17.67, 20.17)	-0.27 (-17.25, 16.69)	-1.06 (-18.46, 17.16)	0.8 (-9.14, 11.08)	-9.31 (-28.62, 10.7)
		PECS	6.01 (-16.15, 30.21)	5.89 (-7.64, 22.56)	5.08 (-16.22, 27.62)	4.1 (-9.08, 20.18)	6.18 (-10.86, 24.51)	-3.97 (-26.83, 20.96)
			PIFB	-0.07 (-23.15, 23.7)	-0.96 (-23.56, 20.88)	-1.83 (-24.06, 20.96)	0.12 (-17.99, 17.92)	-10.02 (-34.13, 14)
				PINB	-0.97 (-23.66, 20.94)	-1.78 (-16.63, 12.98)	0.15 (-18.39, 17.84)	-9.96 (-34.24, 13.96)
					PVB	-0.79 (-21.84, 20.85)	1.06 (-13.79, 16.1)	-9.06 (-31.62, 14.27)
						SAPB	1.89 (-15.29, 18.5)	-8.2 (-31.63, 14.88)
							TEA	-10.09 (-28.79, 9.01)
								TTMPB

Table S6. Head-to-head comparisons of pain score 6h at cough

Control	-25.4 (-88.41, 37.41)	-28.94 (-86.28, 28.11)	-19.9 (-77.06, 37.35)	-12.5 (-53.8, 30.02)	-22.23 (-86.06, 41.38)	-19.81 (-80.96, 41.22)	**-26.18 (-52, -0.38)**	-20.06 (-77.5, 37.47)
	ESPB	-3.5 (-88.46, 81.72)	5.52 (-79.3, 91.11)	12.97 (-62.46, 89.18)	3.24 (-78.48, 84.76)	5.59 (-82.29, 93.29)	-0.72 (-58.11, 56.74)	5.41 (-79.93, 91.04)
		PECS	9 (-71.58, 90.03)	16.55 (-54.09, 87.84)	6.73 (-79.21, 92.54)	9.16 (-74.41, 92.63)	2.79 (-59.9, 65.21)	8.94 (-72.28, 90.04)
			PIFB	7.44 (-63.18, 79.04)	-2.27 (-88.3, 83.21)	0.13 (-83.63, 83.8)	-6.22 (-69.43, 56.53)	-0.19 (-80.82, 80.7)
				PINB	-9.76 (-86.94, 65.86)	-7.37 (-82.08, 65.97)	-13.69 (-63.54, 35.37)	-7.53 (-79.63, 63.3)
					PVB	2.29 (-85.68, 90.52)	-4.01 (-61.98, 54.11)	2.19 (-83.44, 88.38)
						SAPB	-6.35 (-72.59, 59.91)	-0.18 (-83.72, 83.69)
							TEA	6.08 (-56.79, 69.44)
								TTMPB

Table S7. Head-to-head comparisons of pain score 12h at rest

Control	-9.61 (-22.22, 3.51)	-13.71 (-32.63, 5.23)	**-25 (-49.69, -0.2)**	-4.43 (-24.58, 16.1)	-9.22 (-22.28, 3.64)	-13.76 (-32.52, 5.33)	**-13.3 (-23.83, -3.01)**	**-24.43 (-45.32, -3.39)**
	ESPB	-4.08 (-27.23, 18.57)	-15.36 (-43.56, 12.39)	5.17 (-18.95, 29.23)	0.41 (-17.43, 17.46)	-4.12 (-27.17, 18.56)	-3.68 (-18.87, 10.72)	-14.79 (-39.55, 9.59)
		PECS	-11.26 (-42.37, 19.78)	9.26 (-11.17, 30.14)	4.48 (-18.58, 27.32)	-0.06 (-20, 20.19)	0.42 (-21.35, 21.83)	-10.74 (-38.93, 17.63)
			PIFB	20.54 (-11.15, 52.79)	15.8 (-12.24, 43.62)	11.21 (-19.69, 42.5)	11.68 (-15.23, 38.47)	0.58 (-31.82, 32.74)
				PINB	-4.8 (-29.2, 19.09)	-9.3 (-30.13, 11.19)	-8.9 (-32.04, 13.58)	-19.96 (-49.32, 9.14)
					PVB	-4.53 (-27.29, 18.79)	-4.1 (-16.16, 7.9)	-15.24 (-39.7, 9.62)
						SAPB	0.45 (-21.42, 21.75)	-10.67 (-38.87, 17.49)
							TEA	-11.09 (-34.27, 12.42)
								TTMPB

Table S8. Head-to-head comparisons of pain score 12h at cough

Control	-35.25 (-90.54, 20.36)	-21.43 (-71.58, 29.18)	-10.02 (-60.23, 39.89)	-3.84 (-39.83, 32.89)	-20.3 (-53.74, 12.37)	-19.95 (-70.05, 30.14)	**-31.68 (-55.11, -8.24)**	-30.52 (-68.54, 6.97)
	ESPB	13.79 (-61.23, 88.89)	25.21 (-49.44, 100.12)	31.43 (-34.59, 98.16)	14.94 (-44.24, 72.97)	15.25 (-59.51, 89.91)	3.61 (-46.92, 53.84)	4.78 (-62.92, 71.52)
		PECS	11.35 (-60.11, 82.62)	17.52 (-44.17, 79.98)	1.11 (-59.49, 60.43)	1.4 (-69.67, 72.27)	-10.19 (-65.73, 44.91)	-9.07 (-72.43, 53)
			PIFB	6.16 (-55.34, 69.14)	-10.35 (-70.64, 49.63)	-9.94 (-80.94, 61.26)	-21.71 (-76.82, 33.72)	-20.45 (-83.28, 42.23)
				PINB	-16.5 (-66.46, 31.95)	-16.19 (-78.44, 45.26)	-27.87 (-71.59, 15.15)	-26.63 (-79.86, 24.89)
					PVB	0.29 (-59.25, 60.57)	-11.37 (-41.29, 19.41)	-10.15 (-60.03, 39.95)
						SAPB	-11.67 (-66.93, 43.54)	-10.51 (-73.61, 51.75)
							TEA	1.24 (-43.76, 45.09)
								TTMPB

Table S9. Head-to-head comparisons of pain score 24h at rest

Control	-3.37 (-14.76, 8)	1.5 (-18.32, 21.31)	-7.98 (-22.67, 7.65)	-5.42 (-15.47, 4.46)	-10.02 (-30.06, 10.15)	**-9.71 (-14.59, -5.17)**	-0.03 (-17.96, 17.89)
	ESPB	4.88 (-18.03, 27.71)	-4.63 (-23.18, 14.84)	-2.06 (-17.04, 12.75)	-6.64 (-29.81, 16.58)	-6.34 (-18.22, 5.21)	3.32 (-17.96, 24.62)
		PECS	-9.49 (-33.94, 16.07)	-6.91 (-29.16, 15.11)	-11.51 (-39.76, 16.74)	-11.2 (-31.76, 8.97)	-1.56 (-28.21, 25.13)
			PINB	2.57 (-16.19, 20.14)	-2.04 (-27.68, 22.76)	-1.75 (-18.23, 13.59)	7.95 (-15.97, 30.95)
				PVB	-4.63 (-26.88, 17.98)	-4.29 (-14.35, 5.55)	5.37 (-14.99, 26.02)
					SAPB	0.31 (-20.62, 20.74)	9.97 (-16.9, 36.96)
						TEA	9.68 (-8.66, 28.35)
							TTMPB

Table S10. Head-to-head comparisons of pain score 24h at cough

Control	-23.71 (-60.42, 12.93)	3.54 (-31.79, 39.34)	-6.82 (-33.69, 20.98)	-6.64 (-32.31, 18.59)	-9.98 (-45.55, 25.82)	**-16.48 (-27.7, -5.5)**	-13.86 (-42.91, 16.33)
	ESPB	27.25 (-23.86, 78.75)	16.78 (-28.44, 63.12)	16.98 (-26.43, 60.25)	13.73 (-37.58, 65.12)	7.23 (-27.83, 42.21)	9.88 (-36.51, 57.56)
		PECS	-10.39 (-54.92, 34.8)	-10.24 (-54.27, 33.16)	-13.51 (-63.97, 36.91)	-20.04 (-57.57, 16.9)	-17.48 (-63.31, 29.44)
			PINB	0.11 (-37.84, 36.9)	-3.17 (-48.49, 41.61)	-9.63 (-39.63, 19.29)	-7.03 (-47.01, 33.44)
				PVB	-3.24 (-46.99, 40.67)	-9.8 (-35.29, 15.91)	-7.21 (-45.51, 32.74)
					SAPB	-6.49 (-44.21, 30.6)	-3.91 (-49.86, 43.07)
						TEA	2.61 (-28.43, 34.78)
							TTMPB

Table S11. Head-to-head comparisons of pain score 48h at rest

Control	-12.65 (-28.94, 3.38)	-8.81 (-24.63, 7.03)	-9.99 (-32.54, 12.74)	**-8.51 (-15.31, -1.85)**
	ESPB	3.82 (-18.09, 26.06)	2.69 (-24.9, 30.62)	4.14 (-12.06, 20.41)
		PVB	-1.15 (-28.68, 26.45)	0.28 (-15.57, 16.09)
			SAPB	1.48 (-22.34, 24.93)
				TEA

Table S12. Head-to-head comparisons of pain score 48h at cough

Control	-27.25 (-63.09, 7.72)	-9.94 (-42.88, 23)	0.02 (-33.82, 33.5)	**-13.75 (-25.78, -2.67)**
	ESPB	17.26 (-30.74, 66.28)	27.31 (-21.15, 76.15)	13.48 (-19.97, 46.95)
		PVB	9.95 (-37.38, 56.89)	-3.83 (-39.31, 30.66)
			SAPB	-13.76 (-49.75, 21.45)
				TEA

Table S13. Head-to-head comparisons of cumulative morphine consumption 24h

Control	-41.04 (-92.99, 10.71)	-15.61 (-41.45, 10.16)	-6.03 (-58.63, 46.92)	-7.42 (-59.06, 44)	-1.26 (-24.02, 21.48)	-11.76 (-48.08, 24.48)
	ESPB	25.33 (-32.25, 83.38)	35.02 (-38.55, 108.92)	33.53 (-39.18, 106.25)	39.68 (-16.71, 96.51)	29.29 (-34.07, 92.58)
		PINB	9.54 (-49.15, 68.23)	8.13 (-49.75, 65.55)	14.28 (-19.99, 48.88)	3.88 (-40.5, 48.26)
			PVB	-1.39 (-75.45, 71.87)	4.75 (-52.87, 61.91)	-5.78 (-69.66, 58.22)
				SAPB	6.14 (-49.99, 62.51)	-4.22 (-67.75, 58.68)
					TEA	-10.48 (-53.19, 32.36)
						TTMPB

Table S14. Head-to-head comparisons of cumulative morphine consumption 48h

Control	-16.63 (-142.82, 109.93)	-27.35 (-116.43, 62.41)	-6.12 (-133.9, 122.3)	-18.32 (-145.22, 108.98)	-5.9 (-78.82, 67.32)	-38.17 (-165.91, 88.59)
	ESPB	-10.69 (-165.17, 143.46)	10.61 (-168.48, 190.41)	-1.66 (-181.06, 177.14)	10.72 (-134.82, 155.61)	-21.48 (-201.11, 157.25)
		PIFB	21.3 (-135.07, 177.08)	9.12 (-146.76, 165.18)	21.48 (-94.09, 136.02)	-10.76 (-167.04, 143.68)
			PVB	-12.36 (-193.45, 169.17)	0.36 (-147.66, 147.33)	-32.17 (-212.37, 147.78)
				SAPB	12.49 (-134.48, 159.29)	-19.95 (-200.51, 160.57)
					TEA	-32.16 (-179.41, 114.24)
						TTMPB

Table S15. Head-to-head comparisons of need for rescue analgesia

Control	0.48 (0.01, 43.4)	0.02 (0, 2.28)	0.71 (0.01, 63.48)	0.15 (0.01, 1.36)	0.13 (0.01, 2.43)	**0 (0, 0.08)**	**0.1 (0.02, 0.55)**	0.1 (0, 9.62)
	ESPB	0.04 (0, 28.45)	1.5 (0, 865.78)	0.33 (0, 41.81)	0.27 (0, 56.66)	0 (0, 0.21)	0.22 (0, 24.93)	0.21 (0, 123.54)
		PECS	36.02 (0.05, 2.71×10^{4})	7.7 (0.03, 1.41×10^{3})	6.52 (0.02, 1.90×10^{3})	0 (0, 5.7)	5.17 (0.03, 873.95)	5.02 (0.01, 4.00×10^{3})
			PIFB	0.22 (0, 27.44)	0.18 (0, 38.15)	0 (0, 0.15)	0.14 (0, 16.62)	0.14 (0, 87.55)
				PINB	0.85 (0.02, 47.2)	0 (0, 0.6)	0.67 (0.04, 16.18)	0.65 (0, 146.94)
					PVB	0 (0, 0.7)	0.8 (0.05, 12.91)	0.76 (0, 200.93)
						SAPB	**1.04×10^{23} (1.23, 4.18×10^{72})**	9.84×10^{22} (0.95, 4.94×10^{72})
							TEA	0.96 (0.01, 138.35)
								TTMPB

Table S16. Head-to-head comparisons of postoperative nausea and vomiting

Control	0.14 (0, 4.99)	0.67 (0.03, 13.69)	0.13 (0, 3.03)	**0.07 (0, 0.7)**	**0 (0, 0.83)**	0.69 (0.13, 3.66)	0.13 (0, 3.11)
	ESPB	4.97 (0.05, 874.54)	0.99 (0.01, 186.81)	0.5 (0.01, 61.09)	0 (0, 11.72)	5.08 (0.09, 480.87)	0.94 (0.01, 180.97)
		PIFB	0.2 (0, 15.32)	0.1 (0, 4.51)	0 (0, 1.74)	1.04 (0.03, 32.01)	0.19 (0, 15.52)
			PINB	0.51 (0.01, 29.15)	0 (0, 9.26)	5.16 (0.15, 214.39)	0.95 (0.01, 96.71)
				PVB	0 (0, 16.18)	9.96 (0.93, 164.26)	1.85 (0.03, 125.48)
					SAPB	1.07×10^{9} (0.75, 7.14×10^{29})	185050855.54 (0.1, 1.26×10^{29})
						TEA	0.18 (0, 6.79)
							TTMPB

Table S17. Head-to-head comparisons of pruritus

Control	**2.14×10^{-11}(0, 0.08)**	0.16 (0.01, 2.93)	**10.7 (1.05, 246.33)**	0.15 (0.01, 2.92)
	ESPB	**7.43×10^{9} (1.46, 9.96×10^{30})**	5.65×10^{11} (114.29, 6.89×10^{32})	**6.98×10^{9} (1.39, 8.98×10^{30})**
		PINB	**69.32 (1.82, 5812.66)**	0.95 (0.01, 67.77)
			TEA	**0.01 (0, 0.55)**
				TTMPB

Table S18. Head-to-head comparisons of time to tracheal extubation

Control	-75.03 (-236, 86.65)	-72.33 (-306.61, 165.45)	-210.96 (-442.34, 20.5)	-43.11 (-174.89, 89.25)	-128.58 (-274.91, 17.56)	-60.33 (-377.46, 257.01)	**-181.55 (-243.05, -121.33)**	-197.08 (-401.01, 9.47)
	ESPB	3.14 (-281.91, 290.18)	-135.99 (-417.43, 145.21)	31.95 (-176.35, 240.97)	-53.51 (-268.24, 162.72)	14.75 (-340.69, 370.73)	-106.65 (-273.67, 60.75)	-121.76 (-381.41, 139.55)
		PECS	-139.32 (-470.93, 190.75)	29.03 (-209.01, 265.66)	-56.52 (-334.91, 219.88)	11.48 (-291.31, 315.69)	-109.51 (-355.62, 132.66)	-124.54 (-438.79, 188.27)
			PIFB	167.77 (-97.39, 434.81)	82.96 (-191.12, 355.74)	150.92 (-240.7, 542.48)	29.37 (-209.99, 267.81)	14.1 (-293.97, 325.44)
				PINB	-85.47 (-283.33, 111.39)	-17.51 (-321.67, 285.84)	-138.59 (-284.21, 6.43)	-153.32 (-397.33, 89.86)
					PVB	68.12 (-281.34, 417.44)	-53.1 (-199.38, 93.31)	-68.83 (-319.96, 184.19)
						SAPB	-121.16 (-444.94, 201.88)	-136.69 (-512.34, 242.78)
							TEA	-15.43 (-228.76, 199.73)
								TTMPB

Table S19. Head-to-head comparisons of intensive care unit stay

Control	**-18.04 (-29.16, -6.9)**	-11.64 (-32.84, 9.61)	-10.74 (-25.42, 3.75)	-9.16 (-19.79, 1.39)	**-13.83 (-25.76, -2.73)**	-11.63 (-32.8, 9.49)	-4.54 (-10.38, 0.81)	-13.71 (-29.74, 1.38)
	ESPB	6.42 (-17.6, 30.3)	7.31 (-11.06, 25.6)	8.86 (-6.51, 24.2)	4.24 (-12.09, 19.65)	6.42 (-17.49, 30.26)	13.51 (1.48, 25.04)	4.33 (-15.28, 22.97)
		PECS	0.92 (-24.95, 26.59)	2.46 (-15.84, 20.73)	-2.15 (-26.76, 21.38)	0.03 (-18.24, 18.28)	7.12 (-15.12, 28.75)	-2.03 (-28.78, 23.71)
			PIFB	1.55 (-16.43, 19.71)	-3.09 (-21.99, 15.17)	-0.89 (-26.62, 24.83)	6.2 (-9.54, 21.63)	-2.95 (-24.54, 17.87)
				PINB	-4.67 (-20.75, 10.48)	-2.45 (-20.8, 15.8)	4.65 (-7.55, 16.4)	-4.55 (-23.88, 13.59)
					PVB	2.15 (-21.34, 26.65)	9.28 (-2.48, 21.45)	0.12 (-19.28, 19.25)
						SAPB	7.08 (-15.04, 28.79)	-2.06 (-28.68, 23.56)
							TEA	-9.16 (-25.88, 7.07)
								TTMPB

Table S20. Head-to-head comparisons of hospital stay

Control	-0.65 (-2.03, 0.79)	0.6 (-1.23, 2.44)	-0.39 (-1.28, 0.53)	**-0.73 (-1.21, -0.24)**	-1.79 (-3.7, 0.13)
	PIFB	1.26 (-1.1, 3.54)	0.26 (-1.43, 1.91)	-0.08 (-1.61, 1.38)	-1.14 (-3.55, 1.2)
		PINB	-0.99 (-3.03, 1.07)	-1.34 (-3.23, 0.57)	-2.4 (-5.04, 0.26)
			PVB	-0.34 (-1.29, 0.58)	-1.4 (-3.54, 0.7)
				TEA	-1.06 (-3.03, 0.92)
					TTMPB

Table S21. Head-to-head comparisons of mortality

Control	31963.37 (0.42, 1.41×10^{15})	3.3 (0.13, 172.58)	0.73 (0.22, 2.39)
	PIFB	0 (0, 19.64)	0 (0, 2.03)
		PVB	0.22 (0.01, 4.39)
			TEA

Table S22. Assessment of publication bias

Outcome	*P* value of Begg's test	*P* value of Egger's test
Pain score 2-4h at rest	0.235	<0.001
Pain score 2-4h at cough	0.999	0.503
Pain score 6h at rest	0.529	0.653
Pain score 6h at cough	0.583	0.127
Pain score 12h at rest	0.866	0.207
Pain score 12h at cough	0.767	0.519
Pain score 24h at rest	0.750	0.842
Pain score 24h at cough	0.142	0.410
Pain score 48h at rest	0.999	0.589
Pain score 48h at cough	0.631	0.007
Cumulative morphine consumption 24h (mg)	0.511	0.026
Cumulative morphine consumption 48h (mg)	0.466	0.009
Need for rescue analgesia	0.184	0.709
Postoperative nausea and vomiting	0.999	0.853
Pruritus	0.462	0.460
Time to tracheal extubation (minutes)	0.368	<0.001
Intensive care unit stay (hours)	0.001	0.001
Hospital stay (days)	0.835	0.012
Mortality	0.171	0.146

Table S23. Ongoing trials with regional anesthetic techniques for cardiac surgery

Comparison	Register Number	Sponsor/ Collaborators
PVB vs. control	NCT03903367	Beni-Suef University
ESPB	NCT04338984	Campus Bio-Medico University
	NCT04906239	Institutul de Urgen
	NCT04906239	Kahramanmaras Sutcu Imam University
	NCT03825068/NCT03997019	Assiut University
	NCT03541837/NCT03901612	Vinmec Healthcare System
	NCT04447560	Koç University
	ChiCTR2100046786	Fudan University
ESPB vs. PVB	NCT04546113	nstitut Mutualiste Montsouris
PINB	NCT04319588	Campus Bio-Medico University
	NCT04333095	Baylor Research Institute
PIFB	NCT04343105	Tanta University
	ChiCTR2100048244/ ChiCTR2000030609	Nanchang University
TTMPB	NCT04596163	Chinese University of Hong Kong
	NCT04838132	Guangzhou First People's Hospital
	NCT04116554	Fayoum University
	NCT04094532	Ataturk University
	NCT04916418	Turku University Hospital
	ChiCTR2100047755	Nanchang University
ESPB vs. PINB	NCT04090099	Kahramanmaras Sutcu Imam University
SAPB	NCT03237546	NYU Langone Health
	NCT04648774	Jon Bailey

ChiCTR, Chinese Clinical Trial Register; ESPB, erector spinae plane block; NCT, National Clinical Trial; PIFB, pecto-intercostal fascial block; PINB, parasternal intercostal nerve block; PVB, paravertebral block; SAPB, serratus anterior plane block; TTMPB, transversus thoracic muscle plane block.

Figure S1. Risk of bias summary.
(+: low risk of bias; ?: unclear risk of bias; -: high risk of bias.)

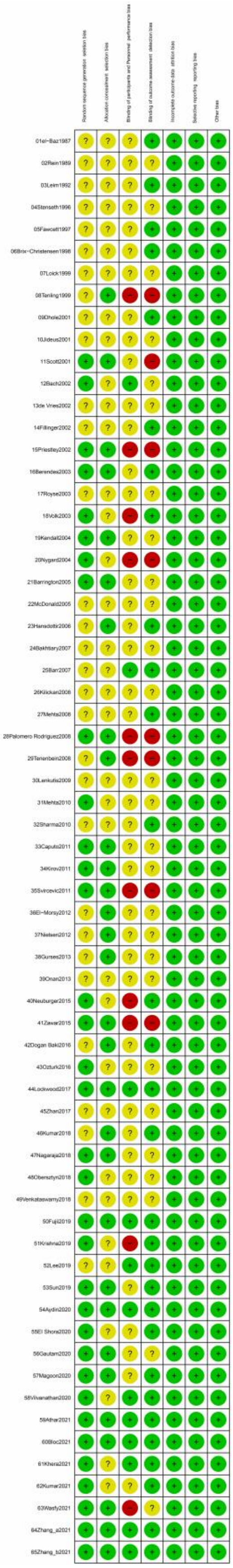

Figure S2. Risk of bias graph.

(+: low risk of bias; ?: unclear risk of bias; -: high risk of bias.)

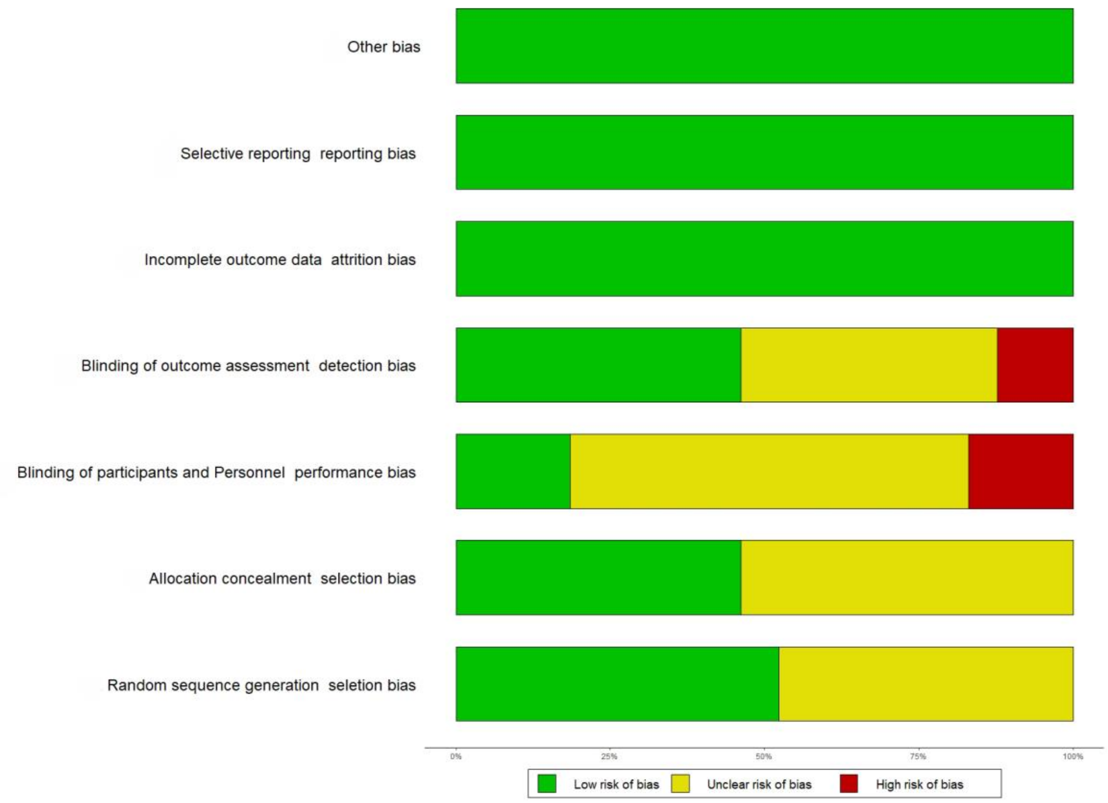

Figure S3. Network plot of eligible comparisons among different regional anesthetic techniques.

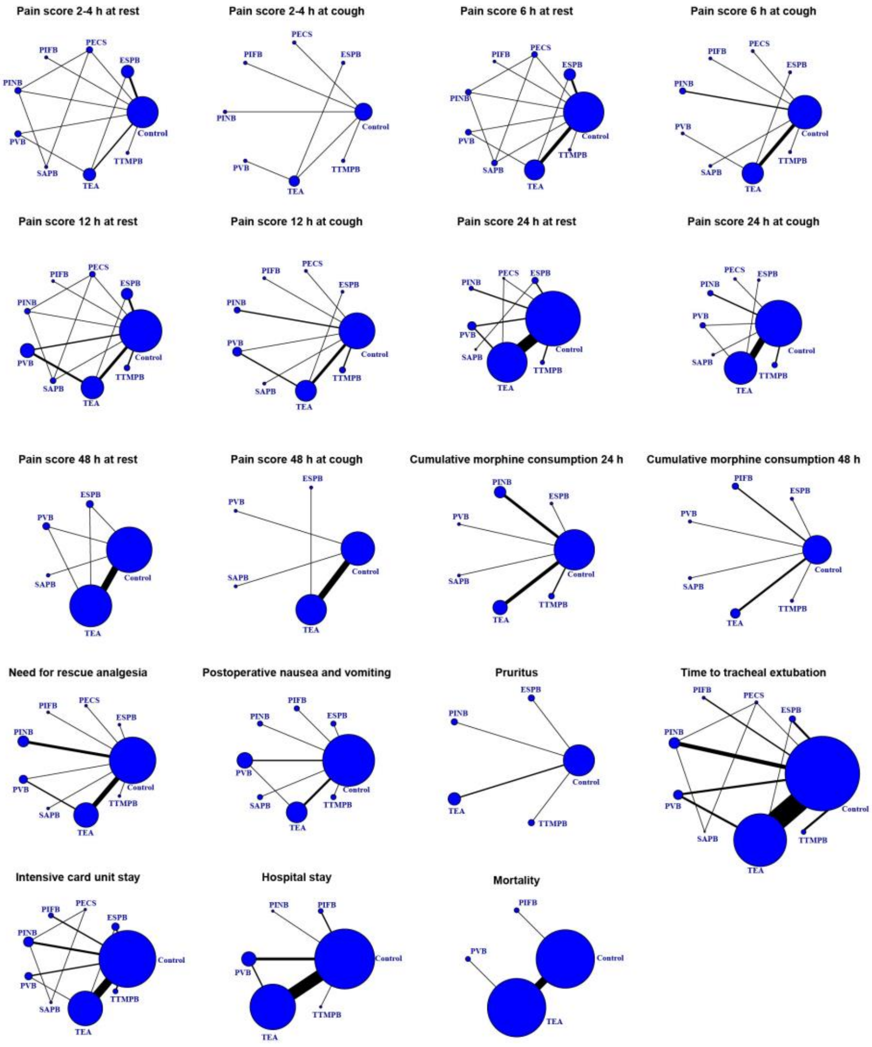

Notes: circles represent the intervention as a node in the network, size of circles corresponds to the number of participants included in each comparison, lines represent direct comparisons using randomized controlled trials (RCTs) and the thickness of lines corresponds to the number of RCTs included in each comparison. ESPB, erector spinae plane block; PECS, pectoral nerve block; PIFB, pecto-intercostal fascial block; PINB, parasternal intercostal nerve block; PVB, paravertebral block; SAPB, serratus anterior plane block; TEA, thoracic epidural analgesia; TTMPB, transversus thoracic muscle plane block.

Figure S4. Inconsistency test of pain scores

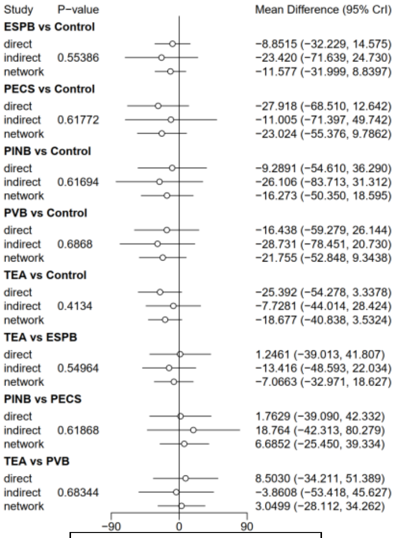

Pain score 2-4h at rest

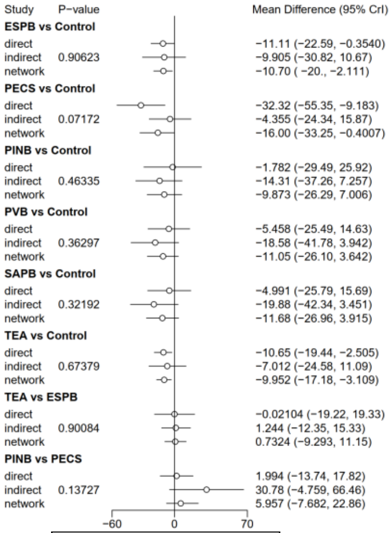

Pain score 6h at rest

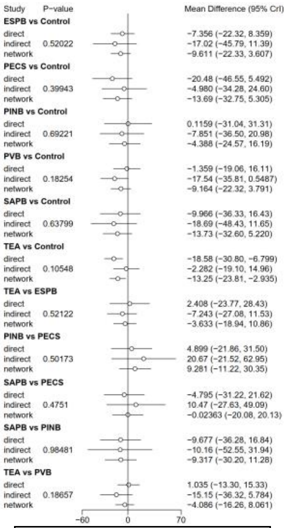

Pain score 12h at rest

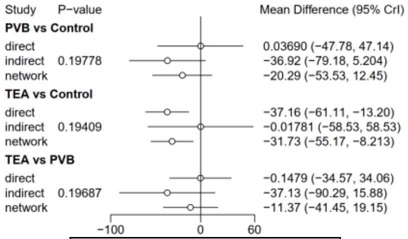

Pain score 12h at cough

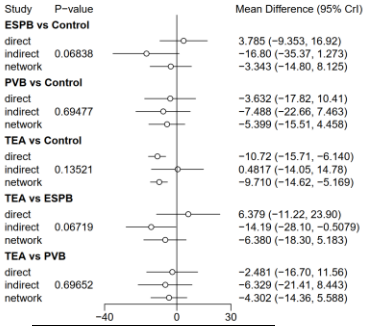

Pain score 24h at rest

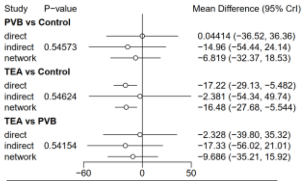

Pain score 24h at cough

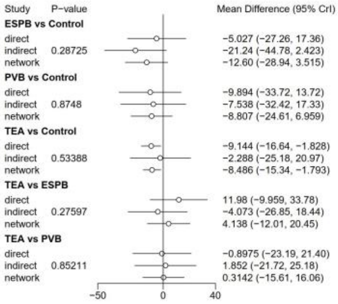

Pain score 48h at rest

Figure S5. Inconsistency test of other outcomes

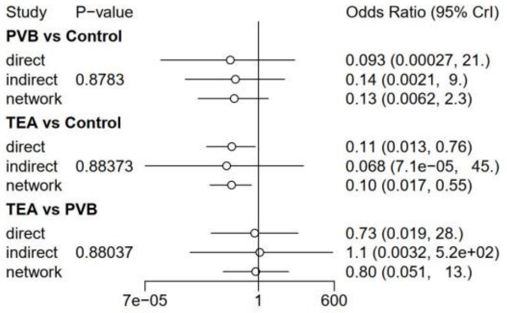

Need for rescue analgesia

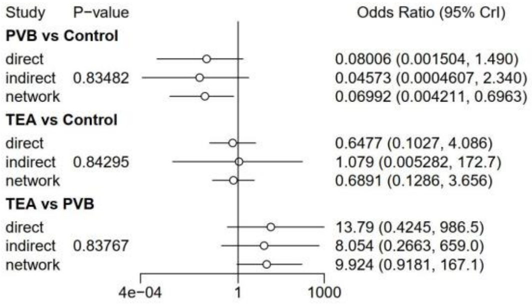

Postoperative nausea and vomiting

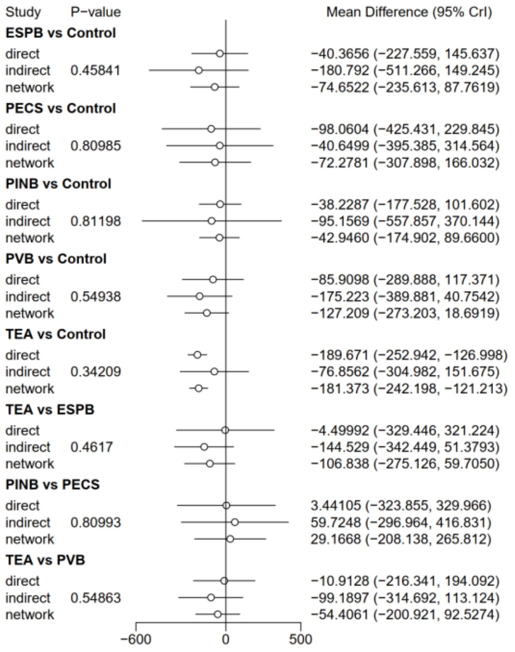

Time to tracheal extubation

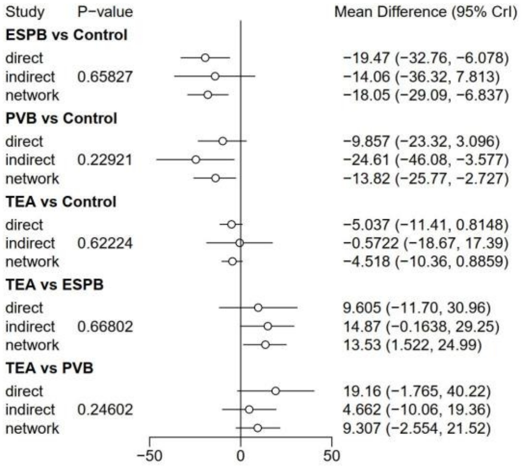

Intensive care unit stay

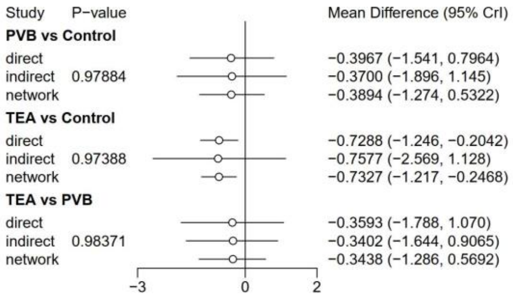

Hospital stay

PRISMA Network Meta-analysis Checklist

**TITLE**			**PAGE**
Title	1	Identify the report as a systematic review *incorporating a network meta-analysis (or related form of meta-analysis).*	**1**
**ABSTRACT**			
Structured summary	2	Provide a structured summary including, as applicable:<br>**Background:** main objectives<br>**Methods:** data sources; study eligibility criteria, participants, and interventions; study appraisal; and *synthesis methods, such as network meta-analysis.*<br>**Results:** number of studies and participants identified; summary estimates with corresponding confidence/credible intervals; *treatment rankings may also be discussed. Authors may choose to summarize pairwise comparisons against a chosen treatment included in their analyses for brevity.*<br>**Discussion/Conclusions:** limitations; conclusions and implications of findings.<br>**Other:** primary source of funding; systematic review registration number with registry name.	**2**
**INTRODUCTION**			
Rationale	3	Describe the rationale for the review in the context of what is already known, *including mention of why a network meta-analysis has been conducted.*	**3**
Objectives	4	Provide an explicit statement of questions being addressed, with reference to participants, interventions, comparisons, outcomes, and study design (PICOS).	**3**
**METHODS**			
Protocol and registration	5	Indicate whether a review protocol exists and if and where it can be accessed (e.g., Web address); and, if available, provide registration information, including registration number.	**4**
Eligibility criteria	6	Specify study characteristics (e.g., PICOS, length of follow-up) and report characteristics (e.g., years considered, language, publication status) used as criteria for eligibility, giving rationale. *Clearly describe eligible treatments included in the treatment network, and note whether any have been clustered or merged into the same node (with justification).*	**4**
Information sources	7	Describe all information sources (e.g., databases with dates of coverage, contact with study authors to identify additional studies) in the search and date last searched.	**4**
Search	8	Present full electronic search strategy for at least one database, including any limits used, such that it could be repeated.	**4**
Study selection	9	State the process for selecting studies (i.e., screening, eligibility, included in systematic review, and, if applicable, included in the meta-analysis).	**4**
Data collection process	10	Describe method of data extraction from reports (e.g., piloted forms, independently, in duplicate) and any processes for obtaining and confirming data from investigators.	**4-5**
Data items	11	List and define all variables for which data were sought (e.g., PICOS, funding sources) and any assumptions and simplifications made.	**4-5**
**Geometry of the network**	**S1**	Describe methods used to explore the geometry of the treatment network under study and potential biases related to it. This should include how the evidence base has been graphically summarized for presentation, and what characteristics were compiled and used to describe the evidence base to readers.	**Figure S3**
Risk of bias within individual studies	12	Describe methods used for assessing risk of bias of individual studies (including specification of whether this was done at the study or outcome level), and how this information is to be used in any data synthesis.	**5**
Summary measures	13	State the principal summary measures (e.g., risk ratio, difference in means). *Also describe the use of additional summary measures assessed, such as treatment rankings and surface under the cumulative ranking curve (SUCRA) values, as well as modified approaches used to present summary findings from meta-analyses.*	**5**
Planned methods of analysis	14	Describe the methods of handling data and combining results of studies for each network meta-analysis. This should include, but not be limited to:<br>• *Handling of multi-arm trials;*<br>• *Selection of variance structure;*<br>• *Selection of prior distributions in Bayesian analyses; and*<br>• *Assessment of model fit.*	**5**
**Assessment of Inconsistency**	**S2**	Describe the statistical methods used to evaluate the agreement of direct and indirect evidence in the treatment network(s) studied. Describe efforts taken to address its presence when found.	**5**
Risk of bias across studies	15	Specify any assessment of risk of bias that may affect the cumulative evidence (e.g., publication bias, selective reporting within studies).	**5**
Additional analyses	16	Describe methods of additional analyses if done, indicating which were pre-specified. This may include, but not be limited to, the following:<br>• Sensitivity or subgroup analyses;<br>• Meta-regression analyses;<br>• *Alternative formulations of the treatment network; and*<br>• *Use of alternative prior distributions for Bayesian analyses (if applicable).*	**NR**
**RESULTS†**			
Study selection	17	Give numbers of studies screened, assessed for eligibility, and included in the review, with reasons for exclusions at each stage, ideally with a flow diagram.	**5-6**
**Presentation of network structure**	**S3**	Provide a network graph of the included studies to enable visualization of the geometry of the treatment network.	**Figure S3**
**Summary of network geometry**	**S4**	Provide a brief overview of characteristics of the treatment network. This may include commentary on the abundance of trials and randomized patients for the different interventions and pairwise comparisons in the network, gaps of evidence in the treatment network, and potential biases reflected by the network structure.	**6**
Study characteristics	18	For each study, present characteristics for which data were extracted (e.g., study size, PICOS, follow-up period) and provide the citations.	**6**
Risk of bias within studies	19	Present data on risk of bias of each study and, if available, any outcome level assessment.	**Supplemental file**
Results of individual studies	20	For all outcomes considered (benefits or harms), present, for each study: 1) simple summary data for each intervention group, and 2) effect estimates and confidence intervals. *Modified approaches may be needed to deal with information from larger networks.*	**6**
Synthesis of results	21	Present results of each meta-analysis done, including confidence/credible intervals. *In larger networks, authors may focus on comparisons versus a particular comparator (e.g. placebo or standard care), with full findings presented in an appendix. League tables and forest plots may be considered to summarize pairwise comparisons.* If additional summary measures were explored (such as treatment rankings), these should also be presented.	**6-7**
**Exploration for inconsistency**	**S5**	7	**7**
Risk of bias across studies	22	Present results of any assessment of risk of bias across studies for the evidence base being studied.	**7**
Results of additional analyses	23	Give results of additional analyses, if done (e.g., sensitivity or subgroup analyses, meta-regression analyses, *alternative network geometries studied, alternative choice of prior distributions for Bayesian analyses,* and so forth).	**NR**
**DISCUSSION**			
Summary of evidence	24	Summarize the main findings, including the strength of evidence for each main outcome; consider their relevance to key groups (e.g., healthcare providers, users, and policy-makers).	**7-9**
Limitations	25	Discuss limitations at study and outcome level (e.g., risk of bias), and at review level (e.g., incomplete retrieval of identified research, reporting bias). *Comment on the validity of the assumptions, such as transitivity and consistency. Comment on any concerns regarding network geometry (e.g., avoidance of certain comparisons).*	**9**
Conclusions	26	Provide a general interpretation of the results in the context of other evidence, and implications for future research.	**10**
**FUNDING**			
Funding	27	Describe sources of funding for the systematic review and other support (e.g., supply of data); role of funders for the systematic review. This should also include information regarding whether funding has been received from manufacturers of treatments in the network and/or whether some of the authors are content experts with professional conflicts of interest that could affect use of treatments in the network.	**NR**

PICOS = population, intervention, comparators, outcomes, study design. † Authors may wish to plan for use of appendices to present all relevant information in full detail for items in this section.

e-Reference

1. el-Baz N, Goldin M. Continuous epidural infusion of morphine for pain relief after cardiac operations. *J Thorac Cardiovasc Surg.* 1987;93(6):878-883.
2. Rein KA, Stenseth R, Myhre HO, Levang OW, Krogstad A. The influence of thoracic epidural analgesia on transcapillary fluid balance in subcutaneous tissue. A study in patients undergoing aortocoronary bypass surgery. *Acta Anaesthesiologica Scandinavica.* 1989;33(1):79-83.
3. Liem TH, Hasenbos MA, Booij LH, Gielen MJ. Coronary artery bypass grafting using two different anesthetic techniques: Part 2: Postoperative outcome. *Journal of Cardiothoracic & Vascular Anesthesia.* 1992;6(2):156-161.
4. Stenseth R, Bjella L, Berg EM, Christensen O, Levang OW, Gisvold SE. Effects of thoracic epidural analgesia on pulmonary function after coronary artery bypass surgery. *European Journal of Cardio-Thoracic Surgery.* 1996;10(10):859-865; discussion 866.
5. Fawcett WJ, Edwards RE, Quinn AC, MacDonald IA, Hall GM. Thoracic epidural analgesia started after cardiopulmonary bypass. Adrenergic, cardiovascular and respiratory sequelae. *Anaesthesia.* 1997;52(4):294-299.
6. Brix-Christensen V, Tonnesen E, Sorensen IJ, Bilfinger TV, Sanchez RG, Stefano GB. Effects of anaesthesia based on high versus low doses of opioids on the cytokine and acute-phase protein responses in patients undergoing cardiac surgery. *Acta Anaesthesiologica Scandinavica.* 1998;42(1):63-70.
7. Loick HM, Schmidt C, Van Aken H, et al. High thoracic epidural anesthesia, but not clonidine, attenuates the perioperative stress response via sympatholysis and reduces the release of troponin T in patients undergoing coronary artery bypass grafting. *Anesthesia & Analgesia.* 1999;88(4):701-709.
8. Tenling A, Joachimsson PO, Tyden H, Wegenius G, Hedenstierna G. Thoracic epidural anesthesia as an adjunct to general anesthesia for cardiac surgery: effects on ventilation-perfusion relationships. *Journal of Cardiothoracic & Vascular Anesthesia.* 1999;13(3):258-264.
9. Dhole S, Mehta Y, Saxena H, Juneja R, Trehan N. Comparison of continuous thoracic epidural and paravertebral blocks for postoperative analgesia after minimally invasive direct coronary artery bypass surgery. *Journal of Cardiothoracic & Vascular Anesthesia.* 2001;15(3):288-292.
10. Jidéus L, Joachimsson PO, Stridsberg M, et al. Thoracic epidural anesthesia does not influence the occurrence of postoperative sustained atrial fibrillation. *Annals of Thoracic Surgery.* 2001;72(1):65-71.
11. Scott NB, Turfrey DJ, Ray DA, et al. A prospective randomized study of the potential benefits of thoracic epidural anesthesia and analgesia in patients undergoing coronary artery bypass grafting. *Anesthesia & Analgesia.* 2001;93(3):528-535.
12. Bach F, Grundmann U, Bauer M, et al. Modulation of the inflammatory response to cardiopulmonary bypass by dopexamine and epidural anesthesia. *Acta Anaesthesiol Scand.* 2002;46(10):1227-1235.
13. de Vries AJ, Mariani MA, van der Maaten JM, Loef BG, Lip H. To ventilate or not after minimally invasive direct coronary artery bypass surgery: the role of epidural anesthesia. *J Cardiothorac Vasc Anesth.* 2002;16(1):21-26.
14. Fillinger MP, Yeager MP, Dodds TM, Fillinger MF, Whalen PK, Glass DD. Epidural anesthesia and analgesia: effects on recovery from cardiac surgery. *Journal of cardiothoracic and vascular anesthesia.* 2002;16(1):15-20.
15. Priestley MC, Cope L, Halliwell R, et al. Thoracic epidural anesthesia for cardiac surgery: the effects on tracheal intubation time and length of hospital stay. *Anesthesia & Analgesia.* 2002;94(2):275-282, table of contents.
16. Berendes E, Schmidt C, Van Aken H, et al. Reversible cardiac sympathectomy by high thoracic epidural anesthesia improves regional left ventricular function in patients undergoing coronary artery bypass grafting: a randomized trial. *Archives of Surgery.* 2003;138(12):1283-1290; discussion 1291.
17. Royse C, Royse A, Soeding P, Blake D, Pang J. Prospective randomized trial of high thoracic epidural analgesia for coronary artery bypass surgery. *Annals of Thoracic Surgery.* 2003;75(1):93-100.

18. Volk T, Dopfmer UR, Schmutzler M, et al. Stress induced IL-10 does not seem to be essential for early monocyte deactivation following cardiac surgery. *Cytokine.* 2003;24(6):237-243.
19. Kendall JB, Russell GN, Scawn ND, Akrofi M, Cowan CM, Fox MA. A prospective, randomised, single-blind pilot study to determine the effect of anaesthetic technique on troponin T release after off-pump coronary artery surgery. *Anaesthesia.* 2004;59(6):545-549.
20. Nygård E, Sorensen LH, Hviid LB, et al. Effects of amiodarone and thoracic epidural analgesia on atrial fibrillation after coronary artery bypass grafting. *Journal of Cardiothoracic & Vascular Anesthesia.* 2004;18(6):709-714.
21. Barrington MJ, Kluger R, Watson R, Scott DA, Harris KJ. Epidural anesthesia for coronary artery bypass surgery compared with general anesthesia alone does not reduce biochemical markers of myocardial damage. *Anesth Analg.* 2005;100(4):921-928.
22. McDonald SB, Jacobsohn E, Kopacz DJ, et al. Parasternal block and local anesthetic infiltration with levobupivacaine after cardiac surgery with desflurane: the effect on postoperative pain, pulmonary function, and tracheal extubation times. *Anesth Analg.* 2005;100(1):25-32.
23. Hansdottir V, Philip J, Olsen MF, Eduard C, Houltz E, Ricksten SE. Thoracic epidural versus intravenous patient-controlled analgesia after cardiac surgery: a randomized controlled trial on length of hospital stay and patient-perceived quality of recovery. *Anesthesiology.* 2006;104(1):142-151.
24. Bakhtiary F, Therapidis P, Dzemali O, et al. Impact of high thoracic epidural anesthesia on incidence of perioperative atrial fibrillation in off-pump coronary bypass grafting: a prospective randomized study. *Journal of Thoracic & Cardiovascular Surgery.* 2007;134(2):460-464.
25. Barr AM, Tutungi E, Almeida AA. Parasternal intercostal block with ropivacaine for pain management after cardiac surgery: a double-blind, randomized, controlled trial. *Journal of cardiothoracic and vascular anesthesia.* 2007;21(4):547-553.
26. Kiliçkan L, Yumuk Z, Bayindir O. The effect of combined preinduction thoracic epidural anaesthesia and glucocorticoid administration on perioperative interleukin-10 levels and hyperglycemia. A randomized controlled trial. *Journal of Cardiovascular Surgery.* 2008;49(1):87-93.
27. Mehta Y, Arora D, Sharma KK, Mishra Y, Wasir H, Trehan N. Comparison of continuous thoracic epidural and paravertebral block for postoperative analgesia after robotic-assisted coronary artery bypass surgery. *Annals of Cardiac Anaesthesia.* 2008;11(2):91-96.
28. Palomero Rodríguez MA, Suarez Gonzalo L, Villar Alvarez F, Varela Crespo C, Moreno Gomez Limon I, Criado Jimenez A. Thoracic epidural anesthesia decreases C-reactive protein levels in patients undergoing elective coronary artery bypass graft surgery with cardiopulmonary bypass. *Minerva Anestesiologica.* 2008;74(11):619-626.
29. Tenenbein PK, Debrouwere R, Maguire D, et al. Thoracic epidural analgesia improves pulmonary function in patients undergoing cardiac surgery. *Canadian Journal of Anaesthesia.* 2008;55(6):344-350.
30. Lenkutis T, Benetis R, Sirvinskas E, Raliene L, Judickaite L. Effects of epidural anesthesia on intrathoracic blood volume and extravascular lung water during on-pump cardiac surgery. *Perfusion.* 2009;24(4):243-248.
31. Mehta Y, Vats M, Sharma M, Arora R, Trehan N. Thoracic epidural analgesia for off-pump coronary artery bypass surgery in patients with chronic obstructive pulmonary disease. *Annals of Cardiac Anaesthesia.* 2010;13(3):224-230.
32. Sharma M, Mehta Y, Sawhney R, Vats M, Trehan N. Thoracic epidural analgesia in obese patients with body mass index of more than 30 kg/m2 for off pump coronary artery bypass surgery. *Annals of Cardiac Anaesthesia.* 2010;13(1):28-33.
33. Caputo M, Alwair H, Rogers CA, et al. Thoracic epidural anesthesia improves early outcomes in patients undergoing off-pump coronary artery bypass surgery: a prospective, randomized, controlled trial. *Anesthesiology.* 2011;114(2):380-390.
34. Kirov MY, Eremeev AV, Smetkin AA, Bjertnaes LJ. Epidural anesthesia and postoperative analgesia with ropivacaine and fentanyl in off-pump coronary artery bypass grafting: a randomized, controlled study. *BMC Anesthesiol.* 2011;11:17.

35. Svircevic V, Nierich AP, Moons KG, et al. Thoracic epidural anesthesia for cardiac surgery: a randomized trial. *Anesthesiology.* 2011;114(2):262-270.
36. El-Morsy GZ, El-Deeb A. The outcome of thoracic epidural anesthesia in elderly patients undergoing coronary artery bypass graft surgery. *Saudi journal of anaesthesia.* 2012;6(1):16-21.
37. Nielsen DV, Bhavsar R, Greisen J, Ryhammer PK, Sloth E, Jakobsen CJ. High thoracic epidural analgesia in cardiac surgery. Part 2--high thoracic epidural analgesia does not reduce time in or improve quality of recovery in the intensive care unit. *Journal of Cardiothoracic & Vascular Anesthesia.* 2012;26(6):1048-1054.
38. Gurses E, Berk D, Sungurtekin H, Mete A, Serin S. Effects of high thoracic epidural anesthesia on mixed venous oxygen saturation in coronary artery bypass grafting surgery. *Med Sci Monit.* 2013;19:222-229.
39. Onan B, Onan IS, Kilickan L, Sanisoglu I. Effects of epidural anesthesia on acute and chronic pain after coronary artery bypass grafting. *Journal of Cardiac Surgery.* 2013;28(3):248-253.
40. Neuburger PJ, Ngai JY, Chacon MM, et al. A Prospective Randomized Study of Paravertebral Blockade in Patients Undergoing Robotic Mitral Valve Repair. *Journal of Cardiothoracic & Vascular Anesthesia.* 2015;29(4):930-936.
41. Zawar BP, Mehta Y, Juneja R, Arora D, Raizada A, Trehan N. Nonanalgesic benefits of combined thoracic epidural analgesia with general anesthesia in high risk elderly off pump coronary artery bypass patients. *Annals of Cardiac Anaesthesia.* 2015;18(3):385-391.
42. Doğan Bakı E, Kavrut Ozturk N, Ayoğlu RU, Emmiler M, Karslı B, Uzel H. Effects of Parasternal Block on Acute and Chronic Pain in Patients Undergoing Coronary Artery Surgery. *Semin Cardiothorac Vasc Anesth.* 2016;20(3):205-212.
43. Ozturk NK, Baki ED, Kavakli AS, et al. Comparison of Transcutaneous Electrical Nerve Stimulation and Parasternal Block for Postoperative Pain Management after Cardiac Surgery. *Pain Res Manag.* 2016;2016:4261949.
44. Lockwood GG, Cabreros L, Banach D, Punjabi PP. Continuous bilateral thoracic paravertebral blockade for analgesia after cardiac surgery: a randomised, controlled trial. *Perfusion.* 2017;32(7):591-597.
45. Zhan Y, Chen G, Huang J, Hou B, Liu W, Chen S. Effect of intercostal nerve block combined with general anesthesia on the stress response in patients undergoing minimally invasive mitral valve surgery. *Experimental and Therapeutic Medicine.* 2017;14(4):3259-3264.
46. Kumar KN, Kalyane RN, Singh NG, et al. Efficacy of bilateral pectoralis nerve block for ultrafast tracking and postoperative pain management in cardiac surgery. *Annals of Cardiac Anaesthesia.* 2018;21(3):333-338.
47. Nagaraja PS, Ragavendran S, Singh NG, et al. Comparison of continuous thoracic epidural analgesia with bilateral erector spinae plane block for perioperative pain management in cardiac surgery. *Annals of Cardiac Anaesthesia.* 2018;21(3):323-327.
48. Obersztyn M, Trejnowska E, Nadziakiewicz P, Knapik P. Evaluation of thoracic epidural analgesia in patients undergoing coronary artery bypass surgery - a prospective randomized trial. *Kardiochir Torakochirurgia Pol.* 2018;15(2):72-78.
49. Venkataswamy M, Ramakrishna PS, Nagaraja PS, Singh NG, Adoni PJ. Efficacy of bilateral continuous paravertebral block for off pump coronary artery bypass surgery. *Journal of Cardiovascular Disease Research.* 2018;9(2):59-62.
50. Fujii S, Roche M, Jones PM, Vissa D, Bainbridge D, Zhou JR. Transversus thoracis muscle plane block in cardiac surgery: a pilot feasibility study. *Regional Anesthesia & Pain Medicine.* 2019;44(5):556-560.
51. Krishna SN, Chauhan S, Bhoi D, et al. Bilateral Erector Spinae Plane Block for Acute Post-Surgical Pain in Adult Cardiac Surgical Patients: A Randomized Controlled Trial. *Journal of Cardiothoracic & Vascular Anesthesia.* 2019;33(2):368-375.
52. Lee CY, Robinson DA, Johnson CA, et al. A Randomized Controlled Trial of Liposomal Bupivacaine Parasternal Intercostal Block for Sternotomy. *Annals of thoracic surgery.* 2019;107(1):128-134.
53. Sun L, Li Q, Wang Q, Ma F, Han W, Wang M. Bilateral thoracic paravertebral block combined with general anesthesia vs. general anesthesia for patients undergoing off-pump

coronary artery bypass grafting: a feasibility study. *BMC Anesthesiology.* 2019;19(1):101.
54. Aydin ME, Ahiskalioglu A, Ates I, et al. Efficacy of Ultrasound-Guided Transversus Thoracic Muscle Plane Block on Postoperative Opioid Consumption After Cardiac Surgery: A Prospective, Randomized, Double-Blind Study. *Journal of Cardiothoracic & Vascular Anesthesia.* 2020;34(11):2996-3003.
55. El Shora HA, El Beleehy AA, Abdelwahab AA, et al. Bilateral Paravertebral Block versus Thoracic Epidural Analgesia for Pain Control Post-Cardiac Surgery: A Randomized Controlled Trial. *Thoracic & Cardiovascular Surgeon.* 2020;68(5):410-416.
56. Gautam S, Pande S, Agarwal A, et al. Evaluation of Serratus Anterior Plane Block for Pain Relief in Patients Undergoing MIDCAB Surgery. *Innovations: Technology and Techniques in Cardiothoracic and Vascular Surgery.* 2020;15(2):148-154.
57. Magoon R, Kaushal B, Chauhan S, Bhoi D, Bisoi A, Khan M. A randomised controlled comparison of serratus anterior plane, pectoral nerves and intercostal nerve block for post-thoracotomy analgesia in adult cardiac surgery. *Indian journal of anaesthesia.* 2020;64(12):1018-1024.
58. Vilvanathan S, Saravanababu MS, Sreedhar R, Gadhinglajkar SV, Dash PK, Sukesan S. Ultrasound-guided Modified Parasternal Intercostal Nerve Block: Role of Preemptive Analgesic Adjunct for Mitigating Poststernotomy Pain. *Anesth Essays Res.* 2020;14(2):300-304.
59. Athar M, Parveen S, Yadav M, et al. A Randomized Double-Blind Controlled Trial to Assess the Efficacy of Ultrasound-Guided Erector Spinae Plane Block in Cardiac Surgery. *J Cardiothorac Vasc Anesth.* 2021:Mar 08;S1053-0770(1021)00211-00211.
60. Bloc S, Perot BP, Gibert H, et al. Efficacy of parasternal block to decrease intraoperative opioid use in coronary artery bypass surgery via sternotomy: a randomized controlled trial. *Reg Anesth Pain Med.* 2021;46(8):671-678.
61. Khera T, Murugappan KR, Leibowitz A, et al. Ultrasound-Guided Pecto-Intercostal Fascial Block for Postoperative Pain Management in Cardiac Surgery: A Prospective, Randomized, Placebo-Controlled Trial. *Journal of Cardiothoracic & Vascular Anesthesia.* 2021;35(3):896-903.
62. Kumar AK, Chauhan S, Bhoi D, Kaushal B. Pectointercostal Fascial Block (PIFB) as a Novel Technique for Postoperative Pain Management in Patients Undergoing Cardiac Surgery. *Journal of Cardiothoracic & Vascular Anesthesia.* 2021;35(1):116-122.
63. Wasfy SF, Kamhawy GA, Omar AH, Abd El Aziz HF. Bilateral continuous erector spinae block versus multimodal intravenous analgesia in coronary bypass surgery. A Randomized Trial. *Egyptian Journal of Anaesthesia.* 2021;37(1):152-158.
64. Zhang Y, Gong H, Zhan B, Chen S. Effects of bilateral Pecto-intercostal Fascial Block for perioperative pain management in patients undergoing open cardiac surgery: a prospective randomized study. *BMC Anesthesiol.* 2021;21(1):175.
65. Zhang Y, Li X, Chen S. Bilateral transversus thoracis muscle plane block provides effective analgesia and enhances recovery after open cardiac surgery. *Journal of Cardiac Surgery.* 2021;36(8):2818-2823.
